# The mechanism of branched-chain amino acid transferases in different diseases: Research progress and future prospects

**DOI:** 10.3389/fonc.2022.988290

**Published:** 2022-09-02

**Authors:** Xiazhen Nong, Caiyun Zhang, Junmin Wang, Peilun Ding, Guang Ji, Tao Wu

**Affiliations:** ^1^ Institute of Digestive Disease, Longhua Hospital, Shanghai University of Traditional Chinese Medicine, Shanghai, China; ^2^ Institute of Interdisciplinary Integrative Medicine Research, Shanghai University of Traditional Chinese Medicine, Shanghai, China

**Keywords:** Branched chain amino transferase (BCAT), Branched-chain amino acids (BCAA), cancer, target, tumor resistance

## Abstract

It is well known that the enzyme catalyzes the first step of branched-chain amino acid (BCAA) catabolism is branched-chain amino transferase (BCAT), which is involved in the synthesis and degradation of leucine, isoleucine and valine. There are two main subtypes of human branched chain amino transferase (hBCAT), including cytoplasmic BCAT (BCAT1) and mitochondrial BCAT (BCAT2). In recent years, the role of BCAT in tumors has attracted the attention of scientists, and there have been continuous research reports that BCAT plays a role in the tumor, Alzheimer’s disease, myeloid leukaemia and other diseases. It plays a significant role in the growth and development of diseases, and new discoveries about this gene in some diseases are made every year. BCAT usually promotes cancer proliferation and invasion by activating the phosphatidylinositol 3-kinase/protein kinase B/mammalian target of rapamycin pathway and activating Wnt/β-catenin signal transduction. This article reviews the role and mechanism of BCAT in different diseases, as well as the recent biomedical research progress. This review aims to make a comprehensive summary of the role and mechanism of BCAT in different diseases and to provide new research ideas for the treatment, prognosis and prevention of certain diseases.

## Graphical Abstract

The mechanism of branched chain amino transferases (BCATs) in related diseases.There are two main subtypes of human branched chain amino transferase (hBCAT), including cytoplasmic BCAT (BCAT1) and mitochondrial BCAT (BCAT2). High expression of BCAT1 promotes gastric cancer, lung cancer, breast cancer, prostate cancer, glioma, hepatocellular carcinoma, urothelial cancer, melanoma, lymphoma, esophageal squamous cell carcinoma, head and neck squamous cell carcinoma, and nasopharyngeal carcinoma. It also aggravates liver disease, inflammatory disease, myeloid leukaemia, and Alzheimer’s disease. Low expression of BCAT1 exacerbates Parkinson’s disease. In contrast, high levels of BCAT1 protect the heart and help treat acute myocardial infarction. BCAT2 overexpression aggravates pancreatic ductal adenocarcinoma. Inhibiting the expression of BCAT can treat the above diseases. BCATs: branched chain amino transferases; hBCAT: human branched chain amino transferase; BCAT1: cytoplasmic BCAT; BCAT2: mitochondrial BCAT. “+” stands for promotion and “-” for inhibition.

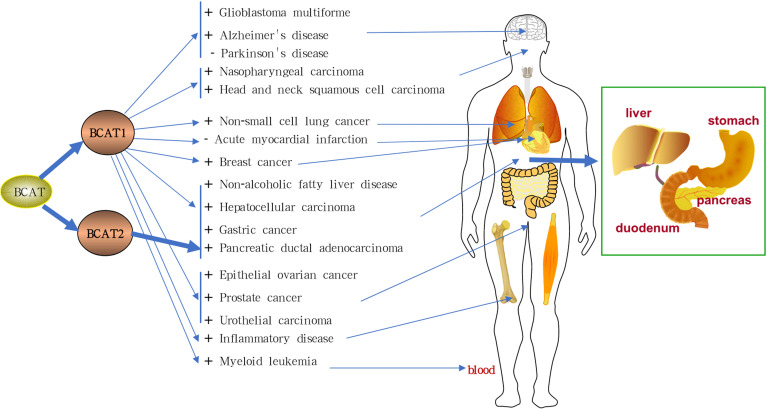

## Highlights

- BCAT plays a role in the tumor, Alzheimer’s disease, myeloid leukaemia and other diseases.
- The expression of BCAT in different cancers is heterogeneous.
- It is beneficial on regulating the level of BCAT for the treatment of cancer, inflammation, liver disease and other diseases.

## 1 Introduction

Branched-chain amino acid transferase (BCAT) are enzymes that catalyze the catabolism of branched-chain amino acids (BCAA), catalyzing the transamination of three BCAA to branched-chain keto acids (BCKA). The branched-chain ketoacid dehydrogenase complex catalyzes the oxidative decarboxylation of branched-chain ketoacids to generate branched-chain acyl-CoA intermediates, which are subsequently involved in different metabolic pathways (1). BCAA metabolism was also accompanied by the reversible amination of α-ketoglutarate (α-KG) to glutamate.

Many types of tumors induce metabolic reprogramming of cells to obtain the energy and nutrients that cells need to proliferate and survive indefinitely. A lot of studies have shown that BCAT was abnormally expressed in different types of tumors, and its expression is either too low or too high, such in breast cancer (BC), urothelial cancer (UC), prostate cancer (PC), lung cancer (LC) and other types of cancer ML (2–4). BCAT is the target of the proto-oncogene c-Myc. BCAT usually promotes cancer proliferation and invasion by activating the phosphatidylinositol 3-kinase (PI3K)/protein kinase B, (PKB; Akt)/mammalian target of rapamycin (mTOR) pathway and Wnt/β-catenin signaling (5). Furthermore, a first report of mutations in the human BCAT2 gene suggests that hypervalinemia and hyperleucine-isoleucinemia may in principle be caused by genetic alterations in the BCAT enzyme protein (6). Every year, new literature reported on the new discoveries of BCAT in diseases such as neoplastic diseases and metabolic diseases. These discoveries constitute a huge information network; involving multiple pathways and mechanisms. By summarizing the extensive literatures on BCAT, we found that BCAT plays different roles in different cancer types and was abnormally activated differently. Therefore, the present review makes a comprehensive summary of the role and mechanism of BCAT in different diseases in order to provide new research ideas for the treatment, prognosis and prevention of certain diseases.

## 2 Structure of BCAT

BCAT is classified as a folded type IV aminotransferase and is a pyridoxal 5’-phosphate (PLP)-dependent enzyme (7). Many PLP-dependent enzymes have been proposed as potential drug targets (8). BCAT usually binds to the cofactor PLP in a dimerized form (9). In addition, redox-active CXXC motifs are found in BCAT, which play roles in protein synthesis, energy metabolism and other pathways, such as glycolytic metabolism (10–12).

**Figure 1:**
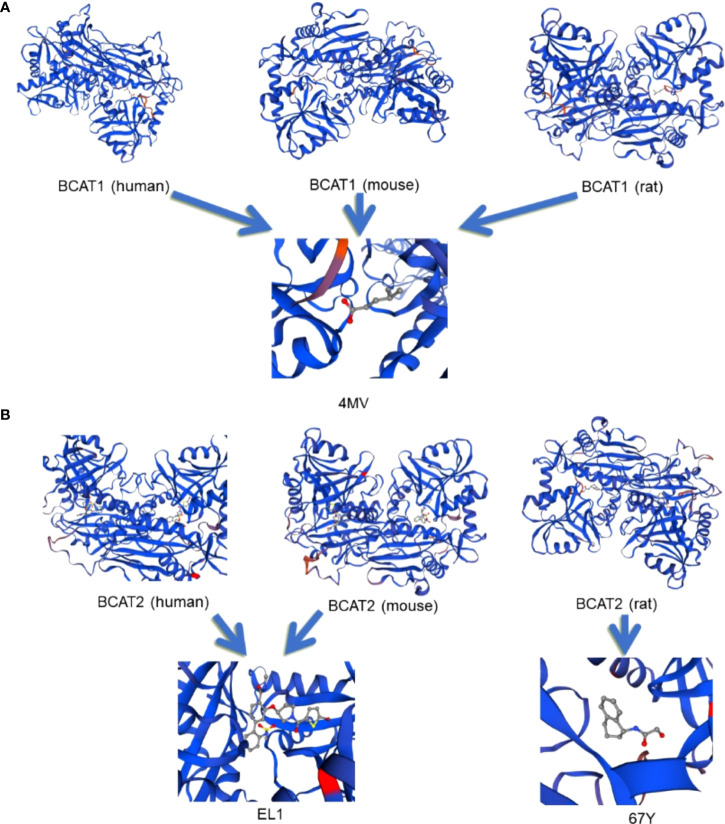
The molecular structures of different types of BCAT protein sequences. Panel **(A)** shows the folded structures of human, rat and mouse BCAT 1 respectively, and their ligand maps, all of which depend on and bind to the cofactor PLP, and the binding ligands are all 4-METHYL VALERIC ACID (4MV). Panel **(B)** shows the folded structures of human, rat, and mouse BCAT2, respectively. Among them, the ligand that binds to human and mouse BCAT2 is 3-({(3R)-1-[(5-bromothiophen-2-yl)carbonyl]pyrrolidin-3-yl}oxy) -N-methyl-2’-[(methylsulfonyl)amino]biphenyl-4-carboxamide (EL1), and the ligand is shown in the figure. The ligand that binds to rat BCAT2 is 2-hydroxy-N-[(1R)-1,2,3,4-tetrahydronaphthalen-1-yl]acetamide (67Y), and the ligand is shown in the figure. All the structural diagrams are drawn on the mapping software SWISS-MODEL. The protein sequence of BCAT is input on the software to export the structural diagram.

**Figure 2:**
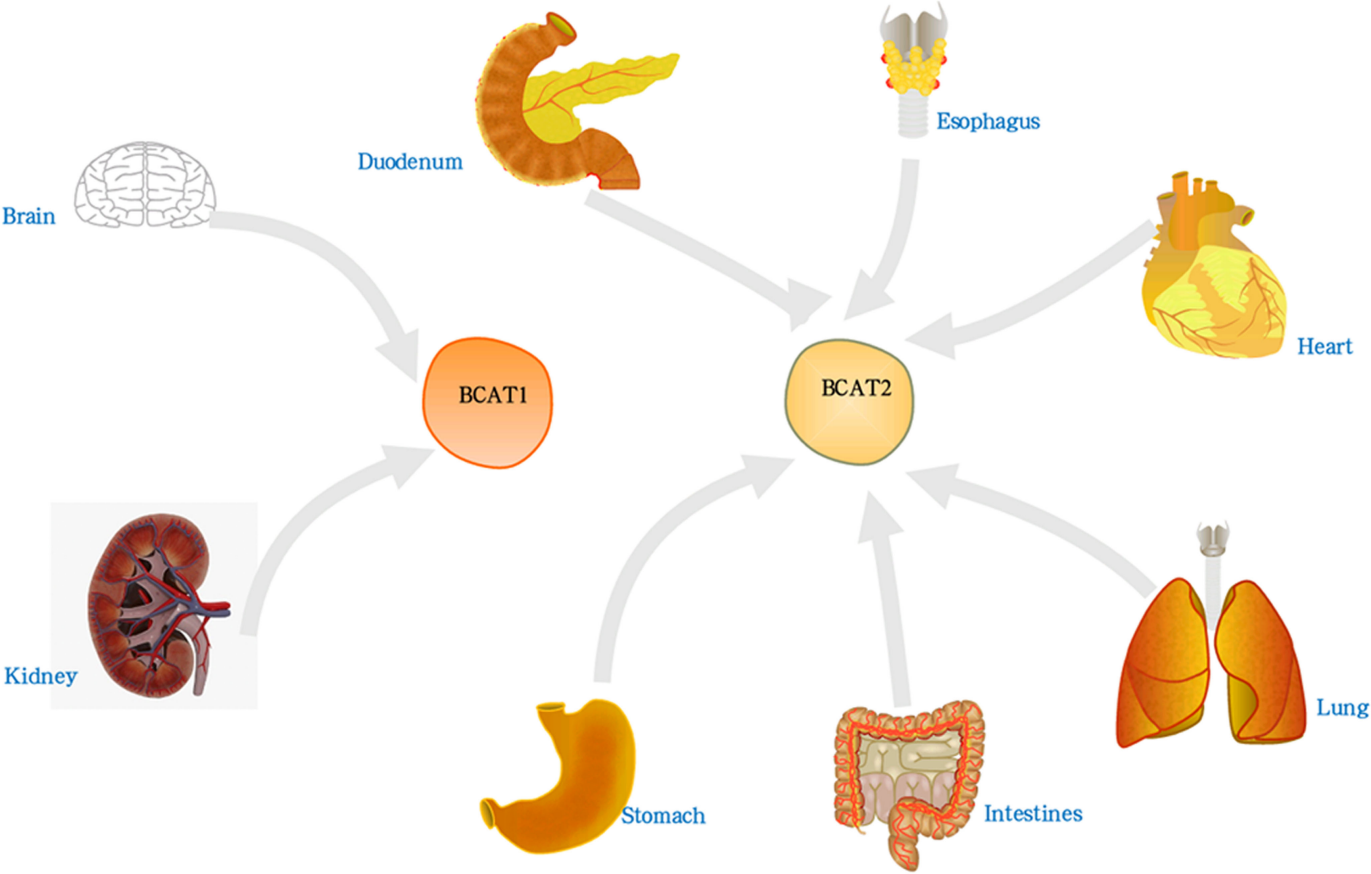
The distribution of BCAT in the human body. BCAT1 was present in brain, kidney and ovarian tissue. BCAT2 was expressed in all organs except liver, such as lung, brain, thorax, etc.

There are two main subtypes of hBCAT including BCAT1 and BCAT2. The protein sequence and molecular structure of BCAT1 and BCAT2 are shown in **Figure 1**, and the corresponding amino acid sequences are shown in the **Supplementary Materials**. BCAT1 is a cytoplasmic aminotransferase encoded by the BCAT1 gene located at 12p12.1 and expressed only in a few tissues, such as the brain, kidney and ovarian tissues (13, 14). BCAT2 is a mitochondrial aminotransferase encoded by the BCAT2 gene located at 19q13.33. It reversibly catalyzes the initial step of the degradation of BCAA to branched-chain acyl-CoA, expressed in many tissues, except mitochondrial protein expressing in all organs other than the liver (15, 16). The approximate distribution of BCAT in the human body is shown in **Figure 2**. Although they have similar substrate specificities, BCAT1 and BCAT2 have different amino acid sequences and exhibit different protein functions (7, 17). The data obtained in the Unified Protein Database and some literature reviews show that the amino acid sequences of BCAT1 and BCAT2 are different. BCAT1 and BCAT2 play different roles in diseases. For example, the expression of BCAT1 was significantly correlated with human epidermal growth factor receptor 2 (HER2+) and luminal B subtypes; the expression of BCAT2 was significantly correlated with luminal A subtypes. BCAT2 was overexpressed in MYC-induced tumors such as Burkitt lymphoma. However, BCAT1 was overexpressed in most cancers such as GC and BC and was involved in various regulatory mechanisms. BCAT2 was ubiquitously expressed in most tissues, whereas BCAT1 was expressed in highly specialized tissues, such as the ovary and the brain. In the brain, BCAT1 was presented in neurons, while BCAT2 was found only in vascular endothelial cells. In the brain, the transamination controlled by BCAT1 is a way of replenishing the glutamate pool and was involved in the release and synthesis of glutamate; while BCAT2 has not been observed to participate in the release of glutamate, some studies have found that it may be involved in glutamate production process (18). Their tissue-specific expression, their roles in different diseases, and their responses to different redox environments suggest that their proteins function differently. In addition, some reports indicated that the regulatory mechanisms and physiological functions of BCAT1 and BCAT2 were significantly different (19, 20).

## 3 BCAT and cancer

### 3.1 BCAT1 and GC

GC is common among many cancer types and ranks among the top three causes of cancer-related death around the world (21). GC has a high mortality rate. Early GC has no obvious symptoms or non-specific symptoms, and the primary tumor is often diagnosed at an advanced stage.

**Figure 3:**
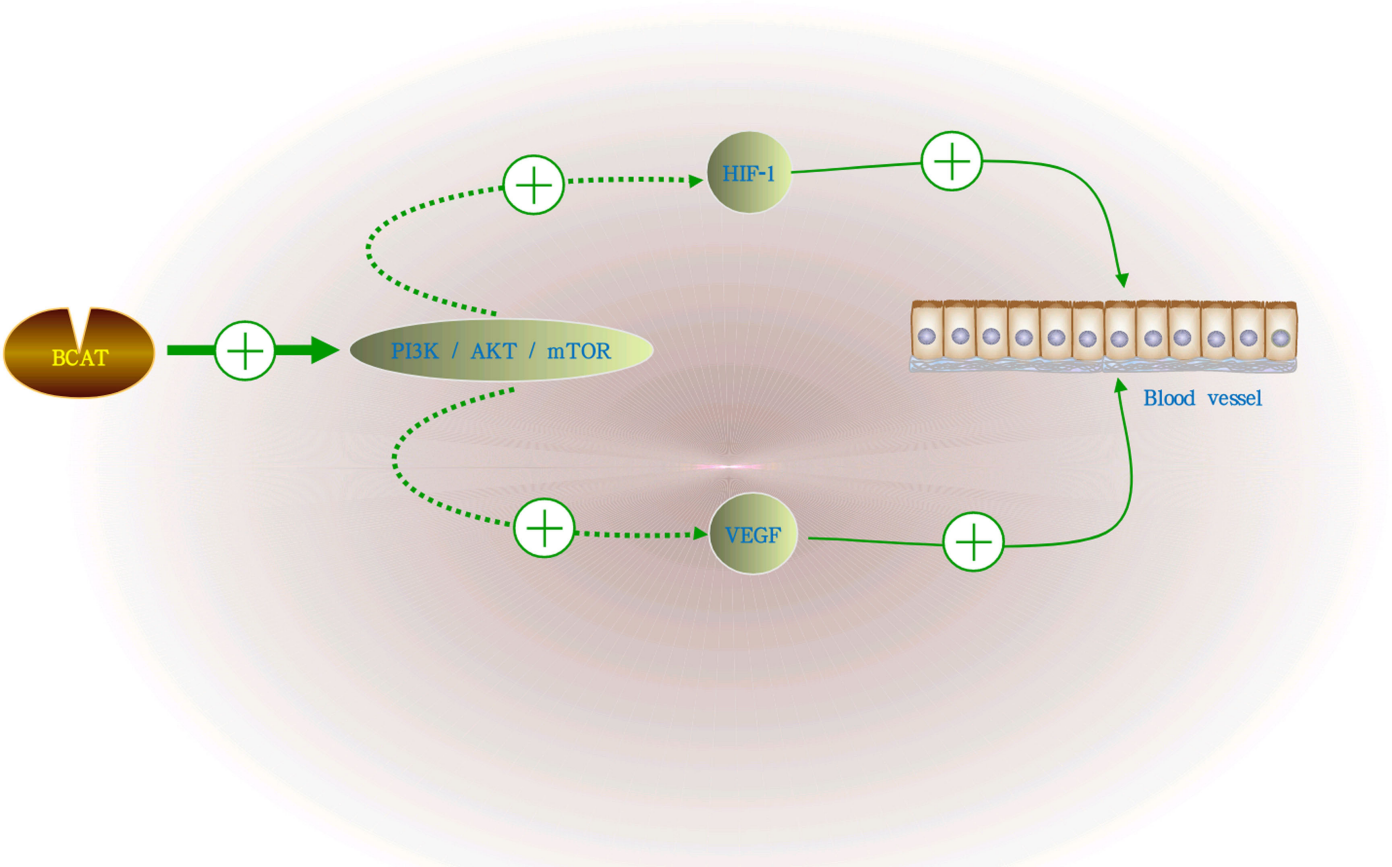
The role of BCAT in GC angiogenesis. BCAT1 activates the PI3K/AKT/mTOR signaling pathway during the growth of GC, and the activation of this pathway can induce the secretion of VEGF, HIF-1 and increase the levels of other angiogenic factors, thereby regulating angiogenesis. “+” means promotion, “-” means inhibition.

Numerous studies have shown that GC is a highly angiogenic cancer (22, 23). In human tumor blood vessels, hypoxia-inducible factor-1 (HIF-1) and vascular endothelial growth factor (VEGF) are the main promoters of tumor angiogenesis. Activation of the PI3K/AKT/mTOR signaling pathway can induce the secretion of VEGF, thereby increasing the levels of other angiogenic factors, such as nitric oxide and angiopoietin, to regulate angiogenesis. The PI3K/AKT/mTOR pathway was closely related to the regulation of BCAT1 and participates in its regulatory mechanism as a downstream pathway of BCAT1. BCAT1 can activate the PI3K/AKT/mTOR pathway during the occurrence and development of GC, thereby promoting proliferation, invasion and angiogenesis (24). It is also shown in **Figure 3**.

Evidence suggests that BCAAs act as nutritional signals to activate and regulate the PI3K/AKT/mTOR signaling pathway, as well as growth factors, by which BCAA affects glucose (Glu) and lipid metabolism, protein synthesis, and gut health (25). As an enzymatic protein in the synthesis and decomposition of BCAA, the transamination of amino groups by BCAA to their respective α-keto acids and glutamate was catalyzed by BCAT1 (26). In addition, Xu et al. (27) found that BCAT1 was positively correlated with Tumor Node Metastasis staging, local invasion, Lauren classification, tumor classification, lymph node metastasis, and distant metastasis of GC. The above findings indicate that BCAT1 seem to affect the proliferation, invasion and angiogenesis of GC from different pathways; and was involved in multiple aspects of the occurrence and development of GC. Evidence of these studies will be helpful research for the therapy of GC ideas.

It was well-known that by inhibiting the PI3K/AKT/mTOR pathway to reverse BCAT1-mediated tumorigenicity to achieve an effective therapeutic effect, the current inhibitors targeting this pathway have been put into early clinical trials. In summary, these findings present that BCAT1 acts as oncogenes in GC progression, and these findings will provide a very cutting-edge idea for the targeted therapy of GC in the future.

### 3.2 BCAT1 and BC

BC ranks among the most common cancers in the world, and it is also the leading type of cancer that causes cancer deaths in women (28). Based on receptor type, BC was categorized into four groups: HER2+, ER-(progesterone receptor) PR-HER2-, A/B (ER and/or progesterone receptor PR positive and triple-negative breast cancer (TNBC). Treatment and prognosis options vary depending on the oncogene. Due to their extreme aggressiveness, invasiveness, and scarcity of molecular therapeutic options, triple-negative BC and HER2+ have the poorest prognoses of all of them (29).

**Figure 4:**
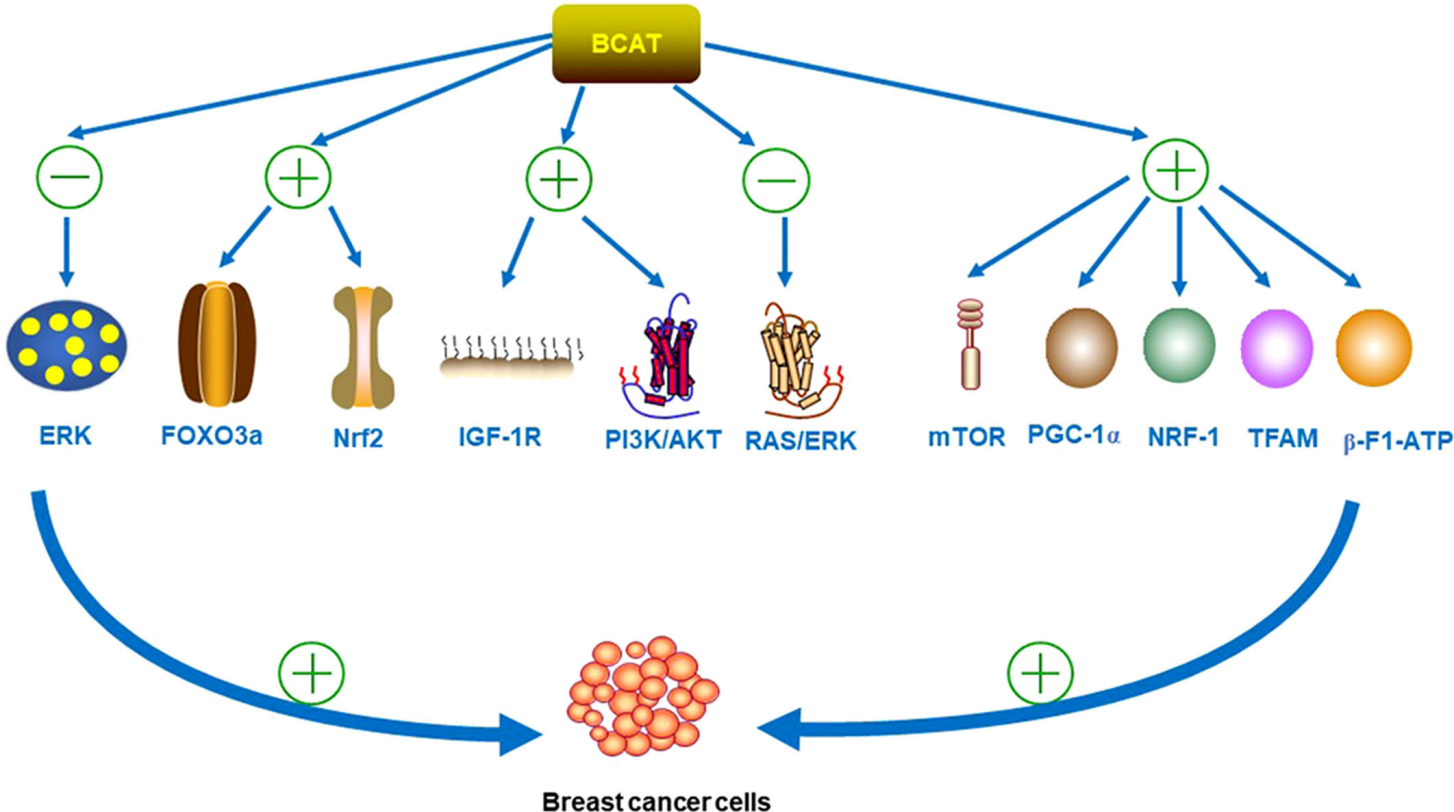
BCAT inhibits the phosphorylation of ERK and upregulates the levels of transcription factors FOXO3a and Nrf2 during the development of BC. BCAT1 also activates the PI3K/AKT axis by stimulating the IGF-1R signaling pathway, while downregulating the RAS/ERK pathway. In addition, BCAT1 also activates mTOR signaling and the expression of PGC-1α, NRF-1, TFAM and β-F1-ATPase, all of which can promote the proliferation, migration and invasion of BC cells. “+” means promotion, “-” means inhibition.

As many types of tumors grow, they reprogram BCAA metabolism to meet their own growth needs (2). Existing experiments have demonstrated that BCAT1 was required for BC growth and development by knockout of the BCAT gene; and was involved in the metastatic spread of BC cells (30). It was found that in terms of proliferation regulation, BCAT1 act as direct target of c-Myc, the activation promoter of metabolic reprogramming in BC. BCAT1 regulates the proliferation, migration and invasion of TNBC cells through the insulin-like growth factor 1 (IGF-1)/insulin PI3K/AKT pathway, inhibits the phosphorylation of extracellular signal-regulated kinase (ERK), and finally upregulates the levels of transcription factors Forkhead box O3 (FOXO3a) and Nuclear Factor erythroid 2-Related Factor 2 (Nrf2) (31, 32). BCAT1 also activates the PI3K/AKT axis by stimulating the insulin-like growth factor 1 receptor (IGF-1R) signaling pathway; while downregulating the renin-angiotensin system (RAS)/ERK pathway (32). In addition, BCAT1 was also involved in the mitochondrial synthesis of cancer cells. Studies have shown that BCAT1 enhance mitochondrial biogenesis by activating mTOR signaling, as well as the expression of Peroxisome proliferator-activated receptor γgamma coactivator 1 alpha (PGC-1α), nuclear respiratory factor-1 (NRF-1), Recombinant Transcription Factor A (TFAM), and β-F1- adenosine-triphosphate (ATP) ase (30). These involved pathways are strong evidence that BCAT1 promote cancer proliferation, and it is shown in **Figure 4**.

Interestingly, the mechanisms of action of BCAT1 and BCAT2 differ in different subtypes of BC, and the expression of BCAT1 was significantly associated with HER2+ and luminal B subtypes; and BCAT2 was significantly associated with luminal A subtype; this finding will be strong support for gene-targeted therapy of different subtypes of BC. In addition, studies have demonstrated that BCAT1 is an independent predictor of TNBC prognosis (33). In conclusion, the role and mechanism of BCAT1 in BC provide a basis for its use as a metabolic imaging biomarker in BC. Although related targeted drugs have not been developed, the BCAT1 gene knockout mouse experiment is feasible, so BCAT1 targeted therapy is a promising strategy for BC.

### 3.3 BCAT2 and PDAC

PDAC is a prevalent cancer diagnosis and is a highly aggressive malignant tumor. It was highly aggressive with no specific symptoms, and had a poor prognosis, resulted in high mortality. Currently, there were limited options for surgical treatment of PDAC, so it was necessary to find its therapeutic targets.

Like other cancers, PDAC induces metabolic reprogramming to meet the needs of its cell proliferation when it occurs, by reprogramming a large amount of synthetic fat and other substances that can provide energy. During cancer growth and survival, the expression of some enzymes changes significantly, such as lipogenic enzymes, malic enzymes, and BCAT, and studying the mechanisms of these enzyme changes, may provide some insights for targeted therapy of pancreatic ductal cancer hope. Studies have found that the carbon source of fatty acid synthesis in an important dependent pathway of pancreatic ductal cancer cells comes from BCAA metabolism (34). Numerous research data show that BCAT2 was overexpressed in PDAC. Lee et al. (34) experimentally demonstrated that BCAT2 gene knockout resulted in a significant reduction in free fatty acid levels in pancreatic ductal cancer cells. In addition, gene knockout of BCAT2 can reduce the oxygen consumption rate of cells, reduce the utilization of some non-essential amino acids and nucleotides, and also affect mitochondrial respiration and reduce BCAA metabolism. These effects can effectively inhibit the malignant proliferation of PDAC cells (35). Furthermore, BCAT2 expression, which was required for collateral lethality caused by deletion of PDAC malic enzyme, was significantly downregulated in pancreatic cancer with increased malice two gene deletion (35, 36). It was exciting news to suggest that there may be new therapeutic directions in the loophole of collateral lethality, but the exact mechanism remains to be further studied.

In addition, BCAT2 has been found to be acetylated on lysine 44 and utilize the ubiquitin-proteasome pathway to accelerate its degradation, resulting in decreased BCAA catabolism, thereby inhibiting pancreatic tumor growth (37). This was an undiscovered mechanism, and the conclusions from these studies suggest that BCAT was promising as a new target for pancreatic ductal cancer gene therapy.

A recent study reveals that BCAT1 was involved in BCAA metabolism loopholes in connective tissue PDACs that were synthetically lethal and cancer-destructive when co-targeting the matrix-BCAT1 and cancer branched-chain alpha-keto acid dehydrogenase (BCKDH) complexes sexual assault (38).

In conclusion, during the growth and proliferation of pancreatic ductal cancer cells, BCAT2 was involved in different pro-proliferative pathways, and inhibition of malignant tumor proliferation by gene knockout of BCAT2 may confer this strategy for targeted therapy of pancreatic ductal cancer. What is worth affirming is that, at present, some patients have alleviated the development of cancer by targeting BCAT2 gene therapy; and achieved specific results.

### 3.4 BCAT and ML

Leukaemia is a life-threatening blood and bone marrow malignant disease caused by abnormal white blood cells in the human body. The oncogenic transformation of hematopoietic stem cells produces leukaemia stem cells (LSCs), divided into acute leukaemia and chronic leukaemia (39, 40). Acute leukaemia is an uncommon malignancy in childhood. Currently, leukaemia was mainly treated clinically through drug intervention and chemotherapy induction. However, the multi-drug resistance generated during the treatment will cause the tumor to recur. It is imperative to find a treatment plan to solve this problem.

#### 3.4.1 BCAT1 and chronic myeloid leukaemia (CML)

Studies have reported that the expression level of BCAT1 increases during the growth and development of BC-CML. BCAT1 synthesizes BCAA from substrates BCKA and glutamate through reversible amination, increasing the pool of BCAA and promoting the proliferation of CML cells. In addition, the study found that the BCAT1 transcript was specifically associated with musashi RNA binding protein 2 (MSI2), a member of the Musashi RNA-binding protein family, and the binding of both parties can regulate the translation of BCAT1 and the activation of mammalian target of rapamycin 1 (mTORC1), which affects the MSI2-BCAT1 axis in the growth and development of CML (41).

These findings support the conclusion that BCAT1 can predict the disease outcome of patients. The use of BCAT1 inhibitors can inhibit the development of the disease, which is of great help for the early diagnosis, intervention and treatment of ML, making targeted therapy of CML a promising solution.

#### 3.4.2 BCAT1 and acute myeloid leukaemia (AML)

It was found that BCAT1 was overexpressed in LSCs of AML. The primary mechanism was that in the BCAA-BCAT1-αKG pathway, BCAT1 stabilized the α protein required for the maintenance of LSCs by regulating the level of intracellular α-KG, limited the activity of α-KG-dependent Recombinant Egl Nine Homolog 1 (EGLN1), reduced the activity of tet methylcytosine dioxygenase 2 (Tet2), these various oncogenic drivers were all designed to promote the rapid growth of malignant tumors (42). Studies have confirmed that when the BCAT1 gene was knocked out, the intracellular α-KG level could be increased, resulted in EGL-1-mediated HIF1 Deoxyribonucleic acid (DNA) protein degradation, which directly leads to cell growth defects and hinders the development of the disease. In addition, the redox-active CXXC motif of BCAT1 had novel antioxidant effects in AML and was involved in the pathogenesis of AML (43). The antioxidant effects of BCAT1 CXXC may contribute to the buffering of intracellular ROS levels in AML cells, thereby affecting disease progression.

In addition, studies on EZH2-deficient leukaemia found that BCAT1 was abnormally activated in EZH2-deficient cancer-initiating cells. The activated BCAT1 cooperated with increased Glu to promote the transamination of BCKA, maintaining the cellular BCAA pool (44). In addition, one study reported that curcumin reduced α-KG levels by inhibiting BCAT1 expression and the mTOR pathway, and finally induced apoptosis in cytarabine-resistant ML cancer cells (45).

In summary, BCAT1 was one of the driving factors of carcinogenesis; and was a critical factor involved in the metabolic process of leukaemia growth and development; for the treatment of leukaemia, BCAT1 gene therapy can be targeted, such as BCAT1 inhibitors, BCAA-BCAT1-α-KG pathway target therapy for isocitrate dehydrogenase (IDH) Wilms Tumor (WT) TET2 WT AML patients with impaired leukaemia stem cell function, etc. These findings can broaden the treatment of leukaemia.

### 3.5 BCAT and other cancers

In addition to the cancers mentioned above, BCAT has also been involved in various other types of cancer. It has been reported that BCAT1 was involved in the proliferation and invasion of epithelial ovarian cancer (46). In the process of ovarian carcinogenesis, gene knockout of BCAT1 can inhibit many oncogenes in the body. The pathways that provide energy and nutrients to cancer cells are also inhibited, such as lipid metabolism, tricarboxylic acid cycle (TCA) cycle, protein synthesis, etc., prolonging the survival time of patients.

In addition, a study reported that BCAT1 as a microRNA (miR)-98-5p target gene promotes cell growth and antagonizes the effect of repressed TMPO-AS1 on colorectal cancer cells (47).

Furthermore, BCAT was also related to the occurrence of UC. Studies have reported that BCAT1 was overexpressed in UC and has implications for the regulation of amino acid metabolism in urinary bladder urothelial cancer (UBUC), which is extremely important for the proliferation, spread and invasion of cancer cells. This indicates that BCAT1 are important in the tumor progression of upper tract urothelial cancer (UTUC) and UBUC (48).

Additionally, a recent study showed the role of mitochondrial BCAT in lymphoma treatment. Deleting the mitochondrial BCAT gene can lead to a considerable accumulation of BCAA that cannot be used for energy or amino acid synthesis, which can ultimately delay the growth of lymphoma tumors (49). This study revealed that mitochondrial BCAT could inhibit tumor development by regulating the concentration of BCAA and it was a therapeutic target that has never been discovered.

Esophageal squamous cell carcinoma (ESCC) was associated with BCAT1. Some studies have reported a fundamental axis in the growth and development of ESCC, namely the methyltransferase 1 (DNMT1)/miR-124/BCAT1 axis. The study found that DNMT1 (Catalytic enzymes involved in the transfer of methyl groups in DNA to specific CpG structures) by mediating the low expression of the tumor suppressor mechanism of h samir-124 3p, targeting its related gene BCAT1, promotes esophagus squamous cells, including the reproduction, spread and invasion of cancer cells (50). Inhibition of DNMT1 and, or BCAT1 activity may inhibit tumor development by blocking this proliferative pathway.

Melanoma is a deadly skin cancer with an extremely high mutation rate (51). Tumor immunotherapy has a low response rate and is prone to drug resistance. Luo et al. (52) proposed that high levels of BCAT1 promote oxidative phosphorylation by increasing the production of acetyl-CoA, thereby enhancing the proliferation and migration of malignant melanoma cells. These studies suggest that BCAT1 as a therapeutic target for malignant melanoma is a novel and promising therapeutic strategy.

In addition, studies have found that high levels of BCAT1 were related to the proliferation and migration of head and neck squamous cell carcinoma (HNSC) cells. It has been reported that in HNSC cells, BCAT1 regulates Glu uptake, and co-regulates the signal transduction of glucose transporters (Glut) family protein glucose transporters 1 (GLUT1) together with the c-Myc gene, thereby regulating the process of cell proliferation and invasion (53). In addition, the mechanism of BCAT1 and c-Myc gene co-regulating the proliferation and invasion of cancer cells was also found in nasopharyngeal carcinoma (NPC). Liu et al. (54) identified a clinically meaningful axis in NPC therapy, the Flotillin 2 (FLOT2)/miR-33b-5p/c-Myc/BCAT1 axis. This study clarified that FLOT2 promotes the high expression of BCAT1 and promotes the development of NPC cells. However, the specific mechanism of BCAT1 in NPC remains to be studied.

In conclusion, BCAT are promising targets for some cancer gene therapy. However, there are still few studies on the role of BCAT in developing these cancers. More specific research was needed to use BCAT as tumor therapy targets and effectively put them into clinical treatment. Only by accurately exploring the mechanism of action of BCAT in tumor development, the crux of the disease can be eliminated.

## 4 BCAT and tumor resistance

### 4.1 BCAT1 and non-small cell lung cancer (NSCLC)

According to the 2021 Global Cancer Report released by the World Health Organization, lung cancer is the most common cancer around the world. NSCLC was a malignant tumor with a high mortality rate. Cancer cells proliferate from primary lung tumor and metastasize to other distant organs, and their malignant growth and metastasis characteristics lead to poor prognosis in NSCLC.

Mao et al. (55) found that BCAT1 was abundantly expressed in metastatic LC cells and regulates the proliferation and spread of cancer cells in the human body. By analyzing a large number of LC literature, we found that BCAT1 promotes the malignant progression of NSCLC from multiple induction pathways. First, C-Myc and cyclinD1 were important oncogenes in the occurrence and development of LC. BCAT1 expression activates Myc and cyclinD1 target genes. Secondly, the study found that the overexpression of BCAT1 up-regulates matrix metalloproteinase 7 (MMP7) and down-regulates E-cadherin in LC. In the process of tumor growth, MMP-7 plays the role in tumor invasion and metastasis. After the transferase up-regulated MMP-7, tumor invasion and metastasis proceeded more rapidly and smoothly. A large number of research data showed that the expression BCAT1 increased, and the expression of c-Myc, cyclinD1 and MMP7 also increased. Not only that, the highly expressed of BCAT1 activates Wnt/β-catenin signaling, and c-Myc, cyclinD1, and MMP7, which are positively regulated by BCAT1, are all targets of the Wnt signaling pathway (56). These pieces of evidence indicate that BCAT1 significantly promotes cell growth and invasion through activating the Wnt signaling pathway and its targets.

In addition, there was a positive response relationship between BCAT1 and poor prognosis in NSCLC. The transcription factor sex determining region Y-box2 (SOX2) was a regulator of cancer stem cell stemness and tumorigenicity, which helps cancer cells evade immune surveillance and resist apoptosis (57). It was well known that BCAT can catalyze the transamination of BCAA into α-KG. The primary mechanism of maintaining the expression of SOX2 is that the high expression of cytoplasmic BCAT reduces the level of α-KG, and the low level of α-KG will reduce the expression of miR-200c, a negative regulator of miR200 family members, thereby affecting the expression of SOX2. In addition, SOX2 regulates the epithelial-mesenchymal transition (EMT) of various cancer types through Wnt signaling, and it has been reported that BCAT1 can induce the EMT process in LC cells. These mechanisms and pathways indicate that the BCAT1 plays a significant role in promoting the rapid proliferation, and spread of LC cells and attacking normal cells to aggravate tumor growth.

In addition, studies have shown that BCAT1 was involved in the occurrence of tyrosine kinase inhibitor resistance in epidermal growth factor receptor LC (58). It was well known that cancer cells undergo metabolic reprogramming under epigenetic regulation, and BCAT1 enhances drug resistance mechanisms by mediating metabolic reprogramming of drug resistance mechanisms. Treatment targeting BCAT1 inhibition may be a potential therapeutic strategy.

These findings herald the important role of BCAT1 in LC. According to existing research reports, BCAT1 independently have a poor prognosis, indicating that BCAT1 can act as a non-small cell Clinical biomarker of malignant behavior in LC (56). In addition, the BCAT1-Wnt/c-Myc axis is a new targeted therapy direction and maybe a hidden strategy for the treatment of LC and its prognosis.

### 4.2 BCAT1 and PC

PC is a widespread malignant tumor. Due to its drug resistance and resistance to chemotherapy, the survival rate of advanced PC patients is still low. The current treatment regimen for patients with advanced PC is to eliminate androgens in the patient’s body. Still, the elimination of androgens is prone to more aggressive prostate tumor recurrence (59).

Billingsley et al. (5) found that BCAT was under expressed in PC tissues. Lower levels of BCAT lead to reduced levels of the hyperpolarized [1-13C]- α-ketoisocaproic acid (KIC) metabolite [1-13C]-Leucine, which has been disrupted by the hyperpolarized [1-13C]-KIC pathway. This finding provides a new idea for assessing prostate tissue. In addition, some studies reported that BCAT1 was a target gene of miR-218, and in this capacity, it was associated with the proliferation, spread and invasion of cancer cells. BCAT1 could increase cell drug resistance, and inhibit the expression of BCAT1 can inhibit tumor development. Inhibition of BCAT1 expression combined with cisplatin may be a potent treatment for PC (60).

### 4.3 BCAT and glioblastoma (GBM)

GBM is a malignant brain tumor with an extremely high recurrence rate due to its poor prognosis and few treatment options, resulting in a slight chance of survival. There are different subtypes of GBM, which are divided into wild-type and mutant based on IDH 1/2 mutation status. IDH1-wild-type GBM is the most progressive GBM type and the highest proportion of primary GBM subtypes; accounting for 90%.

One study discovered that BCAT1 was higher than normal tissue in IDH wild-type tumors using hyperpolarized 13C magnetic resonance spectroscopy (61, 62). Other studies have found that BCAT1 expression was associated with aggressiveness and poor prognosis in IDH1 wild-type gliomas using MR imaging features (63). This suggests that BCAT1 may be a biomarker for tumor subtype classification. Additionally, in previous research reports, BCAT1 has been widely recognized as a new target for the treatment of GBM (64).

Extensive literature has shown that there was significant heterogeneity in brain tumors and that the catabolism of glutamine and glutamate in brain tumors was associated with the proliferation of cancer cells. BCKA and α-KG transglutaminase were derived from BCAA transamination catalyzed by BCATs. GBM induces metabolic reprogramming that alters BCAA metabolism.

A large number of previous research reports have presented that the expression level of BCAT1 plays a crucial role in the occurrence and development of IDH1 wild-type gliomas. BCAT1 was involved in hypoxia, apoptosis, angiogenesis and other processes in GBM (65). The upregulation of BCAT1 in hypoxic GBM is dependent on the hypoxia-inducible factors, participates in cellular metabolic reprogramming and promotes the malignant transformation of tumors (3). In addition, it has been reported that inhibition of BCAT1 in glioma cell lines prevents the proliferation and development of tumor cells by disturbing with tumor energy production and macromolecular synthesis, breakdown and metabolism of BCAAs, and reducing tumor glutamate excretion (66, 67). Besides, the inhibition of BCAT1 expression suppresses glutathione production, which disrupts tumor redox balance. In addition, BCAT1 also plays a role in resistance to tumor therapy. During treatment with bevacizumab, it was found that BCAT1 appears to induce drug resistance by promoting tumor proliferation and glutamate excretion (68). The high expression of BCAT1 enhances the drug resistance of bevacizumab, and inhibiting the expression of BCAI1 is more beneficial to the drug treatment of tumors, suggesting that BCAT1 inhibitor combined with drug therapy may be a potential therapeutic approach. In addition, up-regulation of BCAT1 expression increases glycolysis, providing favorable conditions for tumor proliferation.

In addition, several studies have investigated BCAT2 variant sequences in GBM by mass spectrometry, X-ray crystallography, and proteomic analysis. They have found altered kinetic characteristics after the BCAT2 T186R variant, which may be an indicator of GBM resistance to standard therapy potential factors of the drug (9, 69). BCAT2 T186R can serve as a precise drug target for GBM, but the specific mechanism needs to be further studied.

Furthermore, BCAT1 was associated with muscle wasting in gliomas. Studies have shown that BCAT1 knockout can impair muscle cell growth, reduce mTORC1 and S6K1 phosphorylation levels (PS6K1), and increase ROS synthesis levels (70). This suggests that the role of BCAT1 during muscle cell growth is to activate mTOR signaling and reduce the production of ROS, thereby promoting cell growth.

In conclusion, BCAT plays different roles in the malignant transformation and cell proliferation of glioma. BCAT is an excellent therapeutic target and prognostic factor. The development of its inhibitors may have good clinical potential.

### 4.4 BCAT and Hepatocellular carcinoma (HCC)

HCC is one of the most common cancers worldwide, and its poor prognosis contributes to its extremely high recurrence rate. The main treatment route for HCC is chemotherapy, which is prone to drug resistance. BCAAs are anti-angiogenic in HCC, and supplementation of BCAA in patients can inhibit the development of HCC (71, 72). As an enzyme related to the metabolism of BCAAs, BCAT1 has been shown to regulate the expression of autophagy-related genes in HCC by inducing mTOR-mediated autophagy, and enhancing autophagy in HCC, thereby reducing the sensitivity to chemotherapeutic drugs (52, 73, 74). In addition, BCAT1 was considered to be a direct target of MYC, and MYC overexpression directly activates the BCAT1 promoter, thereby promoting the growth and development of HCC cells. Studies have shown that BCAT1 can resist cisplatin-induced cell death, and inhibiting the expression of BCAT1 can partially block the autophagy response of cancer cells, thereby regulating the sensitivity of cisplatin and reducing tumor drug resistance. These results suggest that BCAT1 may become a clinical marker for HCC prognosis and a pharmacological target for cancer therapy (73).

Ferroptosis is an iron-dependent regulatory cell death type that is associated with a variety of diseases caused by ischemia, such as cancer, and can be used for tumor suppressor-related therapy (75). The development of HCC was closely related to the changes of glutamate levels. Studies have found that BCAT2, as a ferroptosis inhibitor, was involved in the drug-inactivated cystine-glutamate antiporter inhibitor-induced ferroptosis in HCC cells by regulating intracellular glutamate levels (76). Changes in BCAT2 levels were considered sensitive during ferroptosis combined with sorafenib and sulfasalazine treatment. BCAT2 may be involved in treatment as a weather vane for ferroptosis treatment, such as related BCAT2 inhibitors for some clinical treatments.

### 4.5 Common mechanisms of BCAT in tumors

Based on the above-mentioned mechanism of action of BCAT in tumors, we found that BCAT has a common mechanism of action in most tumors. Therefore, we summarize these common mechanisms here to make the “relationship network” between BCAT and tumor clearer.

Various types of tumors can induce cellular metabolic reprogramming, and BCAT, as a key enzyme in the metabolic process of BCAA, was involved in the metabolic reprogramming process. The signaling pathways involved in BCAT in tumor growth mainly include PI3K/AKT/mTOR pathway and Wnt/β-catenin signaling pathway. BCAT in most tumors activates mTOR signaling, such as GC, BC, GBM, ML, HCC. BCAT usually affects glycolysis, fat synthesis, mitochondrial function, BCAA metabolism and other aspects to affect the tumor growth process. For example, the way BCAT1 promotes GC proliferation is to activate the PI3K/AKT/mTOR pathway and induce the secretion of VEGF, thereby increasing the levels of growth factors such as nitric oxide and angiopoietin *in vivo*. The main way that BCAT1 promotes BC proliferation is to stimulate the IGF-1R signaling pathway, activate the PI3K/AKT axis, down-regulate the RAS/ERK pathway, activate mTOR signaling, and enhance mitochondrial production. Tumor types in which BCAT affects proliferation by regulating BCAA metabolism include PDAC, CML, AML, epithelial ovarian cancer, and lymphoma. For example, BCAT1 can synthesize BCAA from substrates BCKA and glutamate through reversible ammoniation, increasing the BCAA pool, thereby promoting CML growth. Activated BCAT1 cooperates with increased Glu to promote transamination of BCKA and maintain cellular BCAA pool levels, thereby affecting the development of EZH2-deficient leukemia.

In addition, BCAT, as a direct target of the target gene C-Myc, was also involved in tumor gene regulation. The mechanism by which BCAT1 and c-Myc genes co-regulate cancer cell proliferation and invasion have been found in many cancers, such as HNSC, NPC, LC and other tumor types. For example, in HNSC, BCAT1 and c-Myc gene co-regulate the signal transduction of GLUT1, thereby regulating the process of cell proliferation and invasion. The FLOT2/miR-33b-5p/c-Myc/BCAT1 axis was found in NPC, which is the axis jointly regulated by BCAT1 and c-Myc genes.

## 5 BCAT and other diseases

### 5.1 BCAT and Alzheimer’s disease (AD)

AD was a metabolic disease associated with people’s age that accounts for more than 60% of all dementias and was characterized by specific neuropathology and impairment of neurotransmitters in the brain (77). One pathogenesis of AD was the accumulation of excitatory neurotransmitter glutamate, which produces neurotoxicity and causes nerve cell death, thereby promoting the development of the disease.

BCAT is a critical enzyme in BCAA catabolism, and catalyzes the reversible amination of BCAAs to generate BCKAs and glutamic acid. Some studies have reported that the overexpression of BCAT in the brain of patients with AD changes the metabolism of BCAAs, and the content of glutamate in the brain of patient increases, which affects the synthesis of neurotransmitters and may cause neuronal toxicity (18, 77–79). In addition, some studies have detected that the accumulation of BCAAs in patients with AD may be caused by the down-regulation of BCAT1 in the brain. The down-regulation of BCAT1 will promote Tau protein phosphorylation. It is mTOR-dependent. That is, BCAT1 plays a role in promoting disease development by activating mTOR signaling (80). The discovery offers potential avenues for treating AD by restricting diets containing BCAA. A research group has demonstrated that the serum BCAT protein and its metabolite glutamate in AD patients were significantly different from those in standard control groups, indicating that BCAT may become the diagnostic pathology of early AD markers (81).

In addition, aggregate accumulation caused by autophagy imbalance is also one of the major causes of neurodegenerative diseases. Several studies have shown that BCAT1 regulates autophagy through the metabolism of BCAAs (82). Some studies have found that the increase of BCAT1 will increase autophagy, and the changes in BCAT1 levels are related to the changes in autophagosome synthesis markers such as LC3 and Beclin. Chain aminotransferases regulate binding to membranes through phosphorylation. Accompanied with reduced Protein Kinase C (PKC) activity, modulation of BCAT1-mediated autophagy may lead to the increased autophagosome synthesis, which may contribute to the accumulation of A-β. In conclusion, BCAT-induced autophagy regulates β-loading through the interdependence of the redox state and PKC phosphorylation, which have an impact on the development of AD (83).

In conclusion, BCAT plays a unique and essential role in early diagnosis and treatment of AD. It can be recognized as a blood marker for the early diagnosis and a potential therapeutic target. Specific clinical applications still need more in-depth research.

### 5.2 BCAT and Parkinson’s disease (PD)

PD is a neurodegenerative disease that causes movement disorders and is more common in older age groups. The main pathological features of the disease are neurodegeneration and loss of substantia nigra neurons, leading to motor dysfunction. The pathogenesis of most PD phenotypes is still unclear, and there is a lack of targeted treatment strategies. Studies have found that the expression of BCAT1 in the substantia nigra of patients with sporadic PD was reduced. The reduction of BCAT1 increases mitochondrial respiration and neuronal damage *in vivo*, resulting in neurodegeneration (84, 85). Changes in the levels of BCAT1 were consistent with age-related changes in PD. This is a potential metabolic pathway, and this discovery provides a new idea for PD treatment strategies, but there are still few studies in this direction. How to eliminate the symptoms of PD patients and cure the disease through this pathway still needs to be more profound research.

### 5.3 BCAT and acute myocardial infarction (AMI)

AMI is a disease with high mortality and morbidity. During the onset, the supply of oxygen and heart blood flow will be drastically reduced, causing myocardial cell damage, and resulting in death. 5-oxo-ETE is a critical metabolite in the process of AMI. The activation of its receptor OXE-R induces an increase in the level of 5-OXO-ETE, which aggravates myocardial injury and promotes myocardial cell apoptosis. Studies have shown that BCAT1, a downstream effector of Oxe-R, plays a significant role in exacerbating cardiomyocyte apoptosis. Elevated levels of BCAT1 can protect against ischemic myocardial injury, and inhibition of Oxe-R can activate the BCAT/mTOR signaling pathway and protect the heart (86). This suggests that targeting BCAT1/BCAT2 is a promising therapeutic direction for the therapy of AMI.

### 5.4 BCAT and Diamond-Blackfan Anemia (DBA)

DBA is a rare congenital disorder and the first human ribosomal disorder characterized by endogenous erythroid hypoplasia (87). Most DBA patients carry ribosomal protein (RP) gene mutations, including RPS19 or RPL11 gene mutations and other gene mutations.

BCAT1 is one of the critical enzymes in the breakdown and production of leu, and leu can improve the anaemia phenotype produced by RPS19 deletion. The study found that BCAT1 in DBA patients has reduced transcript levels and impaired protein translation, and BCAT1 mRNA selectively minimizes the load on cell multimers of DBA patients. In addition, BCAT1 protein expression in the K562 erythroleukemia cell line. The expression level was increased, but the expression was low in hematopoietic stem and progenitor cells, lacking the nuclear factor I family transcription factor Nfix (88). These findings suggest that changes in BCAT1 levels can affect cell proliferation, which in turn affects disease progression, possibly through changes in leu biosynthesis and erythropoiesis.

### 5.5 BCAT and other metabolic diseases

Non-alcoholic fatty liver disease (NAFLD) is a metabolic disease caused by the excessive fat accumulation in liver cells. Because the clinical course of patients with NAFLD varies, there is a significant bottleneck in accurately predicting the development direction of the disease. Research shows that BCAT1 was overexpressed in people with the disease. The highly expressed BCAT1 can convert a large amount of α-KG into glutamate during the metabolic process, resulted in excess glutamate, which leads to the imbalance of α-KG and glutamate in the body (30). This is an important process in liver metabolic disorders, suggesting the feasibility of BCAT1 as a genetic target for this disease.

Insulin resistance (IR), a disorder of Glu metabolism in the body, is one of the most common metabolic complications of obesity and a predictor of type 2 diabetes (T2D) (89). Targeting IR can prevent the malignant development of T2D. Many research reports that change in the metabolic process of BCAAs and the levels of related metabolic enzymes and metabolites are related to IR (71, 90). Increased circulating BCAAs in obese or diabetic patients are positively associated with IR. One study showed that KIC required the push of BCAT2 in inhibiting insulin-stimulated Glut and activated mTORC1 activity, and elevated levels of BCAA and BCAT induced Glut IR in skeletal muscle/muscle cells (91). Studies have shown that the depletion of BCAT2 in cells can effectively attenuate the uptake and utilization of Glu in IR-stimulated myotubes, and the depletion of BCAT2 can eliminate the pro-inflammatory effect of the pro-inflammatory factor KIC (92). In addition, one study showed that BCAT2 was associated with differentiating muscle cells into myotubes, and IR was partly associated with muscle atrophy (11). These findings suggest the potential to treat IR by interfering with BCAT2 levels.

Inflammatory disease (ID) is a defense response of the human body against the invasion of pathogens. It was a common clinical-pathological process. ID can develop into a series of diseases, including cancer. One pathogenesis of chronic ID was the activation of human macrophages stimulated by pro-inflammatory factors, which increases the levels of IRG1 and itaconic acid *in vivo*. Studies have shown that BCAT was overexpressed in macrophages of ID, and BCAT1 regulates macrophage activation through redox-mediated mitochondrial function (93, 94). This was reflected in that inhibiting the expression of BCAT1 could effectively reduce oxygen consumption and glycolysis, and reduced the content of IRG1, itaconic acid and α-KG, thereby reduced the symptoms of inflammation. In addition, inhibition of BCAT1 expression can effectively alleviate the symptoms of sepsis-induced reduction in muscle protein synthesis in systemic inflammation (95). These studies suggest that BCAT1 may be a potential target for targeted therapy of macrophage-dependent ID by indicating BCAT1.

## 6 Others

### 6.1 Influence of BCAT on Chinese hamster ovary (CHO)

CHO cells can produce therapeutic glycoproteins, and the catabolism of BCAAs affects protein synthesis, resulting in low protein production. A study aimed at interfering with BCAT1 and BCAT2 genes by targeting the CRISPR/Cas9 system to explore their possible effects on the expression of recombinant proteins in CHO cells. The experimental results showed that the interference of BCAT1 can promote the cell growth of T2_6 cells producing cherry. In contrast, the interference of BCAT2 only slightly reduced the growth of T2_6 cells, and this effect was cell line and clone dependent (96). This suggests that targeting the BCAT gene has practical significance for recombinant protein synthesis in CHO cells.

Furthermore, a study that metabolically engineered CHO essential amino acids found that endogenous BCAT1 knockout completely abolished the production of inhibitory byproducts in the BCAA catabolic pathway, improving CHO cell growth and productivity (97).

In addition, studies have reported that BCAT1 mediates the self-renewal and pluripotency of mouse embryonic stem cells through Rasal1 and Ras- mitogen-activated protein kinas/ERK signaling pathways (98). These findings have unusual implications for the pathological research and clinical treatment of the disease.

### 6.2 BCAT inhibitors

There is very few research on BCAT inhibitors. Currently, the most commonly used BCAT inhibitor in clinical practice is gabapentin. Gabapentin can inhibit the growth of HCT116 cells and express very low BCAT1 (99). One study showed that combining information from high-throughput screening of HITS and extensive structure-based design can translate HITS into potent BCAT2 inhibitors that could potentially be used to treat obesity (100). The different binding modes of BCAT2 were found through fragment screening, which provided a lot of new ideas for the development of BCAT2 pharmacological inhibitors (101).

## 7 Conclusion

BCAT is one of the critical enzymes in the catabolism process of BCAA, with a redox-active CXXC motif. It plays an essential role in energy formation and conversion, protein and nucleotide synthesis. At the same time, BCAT was also involved in glycolysis, angiogenesis and other processes. In the metabolic reprogramming of tumors, this review aims to make a comprehensive summary of the role and BCAT in different diseases. In order to find breakthroughs in disease treatment, the mechanisms of action, signaling pathways, and therapeutic targeting axes of BCAT in these diseases are comprehensively summarized in **Table 1**. In conclusion, BCAT may be a target for the treatment and prognosis of various diseases, such as LC, BC, AD, IR, etc., which indicates that BCAT gene-targeted therapy will be a promising strategy. In addition, the changes in BCAT1 levels are statistically different in different tumors, and its overexpression in pan-cancer has very obvious prognostic significance (102). There are no clear results so far on the abnormal expression of BCAT in different cancers, and the results found by different researchers are contradictory, which needs to be further studied in the future. Currently, some BCAT-targeted preparations have been put into clinical trials, but the specific mechanism and efficacy still need further research. In addition, the expression levels of BCAT in different disease types are different due to the different metabolic mechanisms of cancer. When studying disease-related treatment plans, the corresponding research directions should be carried out according to the different mechanisms of BCAT. Metabolite biomarkers play an important role in early disease diagnosis, prognosis prediction and targeted treatment. As a potential target for many disease types, BCAT research will make invaluable contributions to human medicine.

**Table 1:** The mechanism of action, signaling pathway and therapeutic targeting axis of BCAT in these diseases.

Diseases	Mechanism	Signaling pathways	Therapeutic targeting axis	Reference
GC	VEGF↑HIF-1↑	PI3K/AKT/mTOR	PI3K/AKT/mTOR	(24–27)
NSCLC	C-Myc↑ cyclinD1↑ MMP-7↑ E-cadherin↓ α-KG↓	Wnt/β-catenin EMT	BCAT1-Wnt/c-Myc	(56–58)
BC	FOXO3a↑ Nrf2↑	IGF-1/Insulin PI3K/AKT, RAS/ERK, mTOR		(30–32)
PC	Leu↓			(5, 60)
PDAC	free fatty acids↑	ubiquitin-proteasome pathway	BCAT1/BCKDH	(34–37)
GBM	Glutathione↑ BCAA catabolism ↑ Glycolysis↑		BCAT2 T186R	(9, 64–69)
CML	BCAA↑	mTORC1 MSI2-BCAT1		(41)
AML	EGLN1↓Tet2↓	BCAA-BCAT1-αKG	BCAA-BCAT1-αKG	(42–45)
HCC	mTOR-mediated autophagy↑			(52, 73–76)
Epithelial ovarian cancer		lipid metabolism TCA cycle protein Synthesis		(46)
UC		UTUC UBUC		(48)
ESCC			DNMT1/miR-124/BCAT1	(50)
melanoma	Acetyl-CoA↑			(51, 52)
HNSC		GLUT1		(53)
NPC			FLOT2/miR-33b-5p/ c-Myc/BCAT1	(54)
AD	Glutamate↑ Tau phosphorylation↓	Mtor Signal Redox state protein kinase C phosphorylation		(77–81, 83)
PD	mitochondrial respiration↑			(84, 85)
AMI		BCAT1/mTOR	BCAT1/BCAT2	(86)
DBA	BCAT1/K562↑ BCAT1/Nfix↓			(88)
NAFLD	α-KG↓Glutamate↑			(30)
IR	KIC↑			(71, 90, 92)
ID	Oxygen consumption ↓ glycolysis ↓ IRG1 ↓ itaconic acid ↓ α-KG ↓			(93–95)

## Author contributions

TW and GJ: conceptualization. XN and CZ: data curation, methodology, software, and writing-original draft preparation. XN, CZ, JW, and PD: visualization and investigation. TW and GJ: supervision and writing- reviewing and editing. All authors contributed to the article and approved the submitted version.

## Funding

This work was supported by the National Natural Science Foundation of China (81873076), the Hundred Talents Program from Shanghai University of Traditional Chinese Medicine and Innovation Project for Undergraduates of Shanghai University of Traditional Chinese Medicine (202210268232).

## Conflict of interest

The authors declare that the research was conducted in the absence of any commercial or financial relationship that could be constructed as a potential conflict of interest.

## Publisher’s note

All claims expressed in this article are solely those of the authors and do not necessarily represent those of their affiliated organizations, or those of the publisher, the editors and the reviewers. Any product that may be evaluated in this article, or claim that may be made by its manufacturer, is not guaranteed or endorsed by the publisher.

## Glossary

**Table:**

AD	Alzheimer's disease
α-KG	α-Ketoglutaric
AKT	(PKB) protein kinase B
AMI	acute myocardial infarction
AML	acute myeloid leukemia
ATP	adenosine-triphosphate
BC	Breast cancer
BCAA	branched-chain amino acid
BCAT	branched-chain amino acid transferase
BCAT1	branched-chain amino acid transferase 1
BCAT2	branched-chain amino acid transferase 2
BCKA	branched-chain keto acid
BCKDH	branched-chain a-ketoacid dehydrogenase
CHO	Chinese hamster ovary
DBA	Diamond-Blackfan Anemia
DNA	deoxyribonucleic acid
DNMT1	methyltransferase 1
EGLN1	recombinant Egl Nine Homolog 1
EMT	epithelial-mesenchymal transition
ERK	extracellular signal-regulated kinase
ESCC	esophageal squamous cell cancer
FLOT2	flotillin 2
FOXO3	Forkhead box O3
GBM	glioblastoma multiforme
GC	gastric cancer
Glu	glucose
Glut	glucose transporters
GLUT1	glucose transporters 1
HCC	hepatocellular carcinoma
HER2+	human epidermal growth factor receptor 2
HIF-1	hypoxia Inducible Factor 1
HNSC	head and neck squamous cell carcinoma
ID	inflammatory disease
IDH	isocitrate dehydrogenase
IGF-1	insulin-like growth factor 1
IGF-1R	insulin-like growth factor 1 receptor
IR	insulin Resistance
KIC	a-ketoisocaproic acid
Leu	leucine
LSCs	leukemia stem cells
miR	microRNA
MMP7	matrix metalloproteinase 7
MSI2	musashi RNA binding protein 2
mTOR	mammalian target of rapamycin
mTORC1	mammalian target of rapamycin 1
NAFLD	non-alcoholic fatty liver disease
NPC	nasopharyngeal carcinoma
NRF-1	nuclear respiratory factor-1
Nrf2	nuclear Factor erythroid 2-Related Factor 2
NSCLC	non-small cell lung cancer
PADC	pancreatic ductal adenocarcinoma
PD	Parkinson's disease
PGC-1α	peroxisome proliferator-activated receptor ggamma coactivator 1α
PI3K	phosphatidy linositol-3-Kinase
PKC	protein kinase C
PLP	pyridoxal 5'-phosphate
PR	progesterone receptor
RAS	renin-angiotensin system
RP	ribosomal protein
SOX2	sex determining region Y-box2
TCA	tricarboxylic acid cycle
T2D	type-2 diabetes
Tet2	tet methylcytosine dioxygenase 2
TFAM	recombinant Transcription Factor A
TNBC	triple negative breast cancer
UBUC	urinary bladder urothelial cancer
UC	urothelial carcinoma
UTUC	upper tract urothelial cancer
VEGF	vascular endothelial growth factor
WT	Wilms Tumor

## References

[1] Adeva-Andany MM Lopez-Maside L Donapetry-Garcia C Fernandez-Fernandez C Sixto-Leal C. Enzymes involved in branched-chain amino acid metabolism in humans. Amino Acids (2017) 49(6):1005–28. doi: 10.1007/s00726-017-2412-7 28324172

[2] Ananieva EA Wilkinson AC. Branched-chain amino acid metabolism in cancer. Curr Opin Clin Nutr Metab Care (2018) 21(1):64–70. doi: 10.1097/MCO.0000000000000430 29211698 PMC5732628

[3] Zhang B Chen Y Shi X Zhou M Bao L Hatanpaa KJ. Regulation of branched-chain amino acid metabolism by hypoxia-inducible factor in glioblastoma. Cell Mol Life Sci (2021) 78(1):195–206. doi: 10.1007/s00018-020-03483-1 32088728 PMC8112551

[4] Dimou A Tsimihodimos V Bairaktari E. The critical role of the branched chain amino acids (BCAAs) catabolism-regulating enzymes, branched-chain aminotransferase (BCAT) and branched-chain alpha-keto acid dehydrogenase (BCKD), in human pathophysiology. Int J Mol Sci (2022) 23(7):4022. doi: 10.3390/ijms23074022 35409380 PMC8999875

[5] Billingsley KL Park JM Josan S Hurd R Mayer D Spielman-Sun E. The feasibility of assessing branched-chain amino acid metabolism in cellular models of prostate cancer with hyperpolarized [1-(13)C]-ketoisocaproate. Magn Reson Imaging (2014) 32(7):791–5. doi: 10.1016/j.mri.2014.04.015 PMC4099288 24907854

[6] Wang XL Li CJ Xing Y Yang YH Jia JP. Hypervalinemia and hyperleucine-isoleucinemia caused by mutations in the branched-chain-amino-acid aminotransferase gene. J Inherit Metab Dis (2015) 38(5):855–61. doi: 10.1007/s10545-015-9814-z 25653144

[7] Toyokawa Y Koonthongkaew J Takagi H. An overview of branched-chain amino acid aminotransferases: functional differences between mitochondrial and cytosolic isozymes in yeast and human. Appl Microbiol Biotechnol (2021) 105(21-22):8059–72. doi: 10.1007/s00253-021-11612-4 34622336

[8] Amorim Franco TM Favrot L Vergnolle O Blanchard JS. Mechanism-based inhibition of the mycobacterium tuberculosis branched-chain aminotransferase by d- and l-cycloserine. HHS Public Access (2017) 12(5):1235–44. doi: 10.1021/acschembio.7b00142. Department of Biochemistry, Albert Einstein College of Medicine, 1300 Morris Park Avenue, Bronx, New York 10461, United States. PMC5834943 28272868

[9] Mehaffey MR Sanders JD Holden DD Nilsson CL Brodbelt JS. Multistage ultraviolet photodissociation mass spectrometry to characterize single amino acid variants of human mitochondrial BCAT2. Anal Chem (2018) 90(16):9904–11. doi: 10.1021/acs.analchem.8b02099 PMC6323636 30016590

[10] Ananieva EA Patel CH Drake CH Powell JD Hutson SM. Cytosolic branched chain aminotransferase (BCATc) regulates mTORC1 signaling and glycolytic metabolism in CD4+ T cells. J Biol Chem (2014) 289(27):18793–804. doi: 10.1074/jbc.M114.554113 PMC4081922 24847056

[11] Dhanani ZN Mann G Adegoke OAJ. Depletion of branched-chain aminotransferase 2 (BCAT2) enzyme impairs myoblast survival and myotube formation. Physiol Rep (2019) 7(23):e14299. doi: 10.14814/phy2.14299 31833233 PMC6908738

[12] Hindy MEL Conway ME. Redox-regulated, targeted affinity isolation of NADH-dependent protein interactions with the branched chain aminotransferase proteins. Methods Mol Biol (2019) 1990:151–63. doi: 10.1007/978-1-4939-9463-2_13 31148070

[13] Sweatt AJ Garcia-Espinosa MA Wallin R Hutson SM. Branched-chain amino acids and neurotransmitter metabolism: expression of cytosolic branched-chain aminotransferase (BCATc) in the cerebellum and hippocampus. J Comp Neurol (2004) 477(4):360–70. doi: 10.1002/cne.20200 15329886

[14] Bixel MG Shimomura Y Hutson S Hamprecht B. Distribution of key enzymes of branched-chain amino acid metabolism in glial and neuronal cells in culture. Volume (2001) 49(3):407–18. doi: 10.1177/002215540104900314 11181743

[15] Knerr I Colombo R Urquhart J Morais A Merinero B Oyarzabal A. Expanding the genetic and phenotypic spectrum of branched-chain amino acid transferase 2 deficiency. J Inherit Metab Dis (2019) 42(5):809–17. doi: 10.1002/jimd.12135 31177572

[16] DeSantiago S Torres N Hutson S Tovar AR. Induction of expression of branched-chain aminotransferase and alpha-keto acid dehydrogenase in rat tissues during lactation. Kluwer Acad (2001) 501:93–9. doi: 10.1007/978-1-4615-1371-1_11 11787736

[17] Conway ME. Emerging moonlighting functions of the branched-chain aminotransferase proteins. Antioxid Redox Signal (2021) 34(13):1048–67. doi: 10.1089/ars.2020.8118 32635740

[18] Hull J Hindy ME Kehoe PG Chalmers K Love S Conway ME. Distribution of the branched chain aminotransferase proteins in the human brain and their role in glutamate regulation. J Neurochem (2012) 123(6):997–1009. doi: 10.1111/jnc.12044 23043456

[19] Kingsbury JM Sen ND Cardenas ME. Branched-chain aminotransferases control TORC1 signaling in saccharomyces cerevisiae. PLoS Genet (2015) 11(12):e1005714. doi: 10.1371/journal.pgen.1005714 26659116 PMC4684349

[20] Costanzo M VanderSluis B Koch EN Baryshnikova A Pons C Tan G. A global genetic interaction network maps a wiring diagram of cellular function. Science (2016) 353(6306):aaf1420. doi: 10.1126/science.aaf1420 27708008 PMC5661885

[21] Venerito M Vasapolli R Rokkas T Malfertheiner P. Gastric cancer: epidemiology, prevention, and therapy. Helicobacter (2018) 23 Suppl 1:e12518. doi: 10.1111/hel.12518 30203589

[22] Chakroborty D Sarkar C Mitra RB Banerjee S Dasgupta PS Basu S. Depleted dopamine in gastric cancer tissues: Dopamine treatment retards growth of gastric cancer by inhibiting angiogenesis. Clin Cancer Res (2004) 10(13):4349–56. doi: 10.1158/1078-0432.CCR-04-0059 15240521

[23] Capuano A Andreuzzi E Pivetta E Doliana R Favero A Canzonieri V. The probe based confocal laser endomicroscopy (pCLE) in locally advanced gastric cancer: A powerful technique for real-time analysis of vasculature. Front Oncol (2019) 9:513. doi: 10.3389/fonc.2019.00513 31263680 PMC6584847

[24] Shu X Zhan PP Sun LX Yu L Liu J Sun LC. BCAT1 activates PI3K/AKT/mTOR pathway and contributes to the angiogenesis and tumorigenicity of gastric cancer. Front Cell Dev Biol (2021) 9:659260. doi: 10.3389/fcell.2021.659260 34164393 PMC8215359

[25] Hassan B Akcakanat A Holder AM Meric-Bernstam F. Targeting the PI3-kinase/Akt/mTOR signaling pathway. Surg Oncol Clin N Am (2013) 22(4):641–64. doi: 10.1016/j.soc.2013.06.008 PMC3811932 24012393

[26] Neinast M Murashige D Arany Z. Branched chain amino acids. Annu Rev Physiol (2019) 81:139–64. doi: 10.1146/annurev-physiol-020518-114455 PMC6536377 30485760

[27] Xu Y Yu W Yang T Zhang M Liang C Cai X. Overexpression of BCAT1 is a prognostic marker in gastric cancer. Hum Pathol (2018) 75:41–6. doi: 10.1016/j.humpath.2018.02.003 29447920

[28] Oktyabri D Ishimura A Tange S Terashima M Suzuki T. DOT1L histone methyltransferase regulates the expression of BCAT1 and is involved in sphere formation and cell migration of breast cancer cell lines. Biochimie (2016) 123:20–31. doi: 10.1016/j.biochi.2016.01.005 26783998

[29] Thewes V Simon R Hlevnjak M Schlotter M Schroeter P Schmidt K. The branched-chain amino acid transaminase 1 sustains growth of antiestrogen-resistant and ERalpha-negative breast cancer. Oncogene (2017) 36(29):4124–34. doi: 10.1038/onc.2017.32 28319069

[30] Wegermann K Henao R Diehl AM Murphy SK Abdelmalek MF Moylan CA. Branched chain amino acid transaminase 1 (BCAT1) is overexpressed and hypomethylated in patients with non-alcoholic fatty liver disease who experience adverse clinical events: A pilot study. PLoS One (2018) 13(9):e0204308. doi: 10.1371/journal.pone.0204308 30265706 PMC6161885

[31] Shafei MA Flemban A Daly C Kendrick P White P Dean S. Differential expression of the BCAT isoforms between breast cancer subtypes. Breast Cancer (2021) 28(3):592–607. doi: 10.1007/s12282-020-01197-7 33367952 PMC8065012

[32] Shafei MA Forshaw T Davis J Flemban A Qualtrough D Dean S. BCATc modulates crosstalk between the PI3K/Akt and the Ras/ERK pathway regulating proliferation in triple negative breast cancer. Oncotarget (2020) 11(12):1971–87. doi: 10.18632/oncotarget.27607 PMC7260123 32523652

[33] Song Y Zhao B Xu Y Ren X Lin Y Zhou L. Prognostic significance of branched-chain amino acid transferase 1 and CD133 in triple-negative breast cancer. BMC Cancer (2020) 20(1):584. doi: 10.1186/s12885-020-07070-2 32571264 PMC7310042

[34] Lee JH Cho YR Kim JH Kim J Nam HY Kim SW. Branched-chain amino acids sustain pancreatic cancer growth by regulating lipid metabolism. Exp Mol Med (2019) 51(11):1–11. doi: 10.1038/s12276-019-0350-z PMC6884453 31784505

[35] Li JT Yin M Wang D Wang J Lei MZ Zhang Y. BCAT2-mediated BCAA catabolism is critical for development of pancreatic ductal adenocarcinoma. Nat Cell Biol (2020) 22(2):167–74. doi: 10.1038/s41556-019-0455-6 32029896

[36] Dey P Baddour J Muller F Wu CC Wang H Liao WT. Genomic deletion of malic enzyme 2 confers collateral lethality in pancreatic cancer. Nature (2017) 542(7639):119–23. doi: 10.1038/nature21052 PMC5398413 28099419

[37] Lei MZ Li XX Zhang Y Li JT Zhang F Wang YP. Acetylation promotes BCAT2 degradation to suppress BCAA catabolism and pancreatic cancer growth. Signal Transduct Target Ther (2020) 5(1):70. doi: 10.1038/s41392-020-0168-0 32467562 PMC7256045

[38] Zhu Z Achreja A Meurs N Animasahun O Owen S Mittal A. Tumour-reprogrammed stromal BCAT1 fuels branched-chain ketoacid dependency in stromal-rich PDAC tumours. Nat Metab (2020) 2(8):775–92. doi: 10.1038/s42255-020-0226-5 PMC7438275 32694827

[39] Nemkov T D'Alessandro A Reisz JA. Metabolic underpinnings of leukemia pathology and treatment. Cancer Rep (Hoboken) (2019) 2(2):e1139. doi: 10.1002/cnr2.1139 32721091 PMC7941580

[40] Juliusson G Hough R. Leukemia. Prog Tumor Res (2016) 43:87–100. doi: 10.1159/000447076 27595359

[41] Hattori A Tsunoda M Konuma T Kobayashi M Nagy T Glushka J. Cancer progression by reprogrammed BCAA metabolism in myeloid leukaemia. Nature (2017) 545(7655):500–4. doi: 10.1038/nature22314 PMC5554449 28514443

[42] Raffel S Falcone M Kneisel N Hansson J Wang W Lutz C. BCAT1 restricts alphaKG levels in AML stem cells leading to IDHmut-like DNA hypermethylation. Nature (2017) 551(7680):384–8. doi: 10.1038/nature24294 29144447

[43] Hillier J Allcott GJ Guest LA Heaselgrave W Tonks A Conway ME. The BCAT1 CXXC motif provides protection against ROS in acute myeloid leukaemia cells. Antioxidants (Basel) (2022) 11(4):683. doi: 10.3390/antiox11040683 35453368 PMC9030579

[44] Gu Z Liu Y Cai F Patrick M Zmajkovic J Cao H. Loss of EZH2 reprograms BCAA metabolism to drive leukemic transformation. Cancer Discovery (2019) 9(9):1228–47. doi: 10.1158/2159-8290.CD-19-0152 PMC6726547 31189531

[45] Tseng YH Yang RC Chiou SS Shieh TM Shih YH Lin PC. Curcumin induces apoptosis by inhibiting BCAT1 expression and mTOR signaling in cytarabineresistant myeloid leukemia cells. Mol Med Rep (2021) 24(2):565. doi: 10.3892/mmr.2021.12204 34109436 PMC8201441

[46] Wang ZQ Faddaoui A Bachvarova M Plante M Gregoire J Renaud MC. BCAT1 expression associates with ovarian cancer progression: possible implications in altered disease metabolism. Oncotarget (2015) 6(31):31522–43. doi: 10.18632/oncotarget.5159 PMC4741622 26372729

[47] Ye J Yan Y Xin L Liu J Tang T Bao X. Long non-coding RNA TMPO-AS1 facilitates the progression of colorectal cancer cells *via* sponging miR-98-5p to upregulate BCAT1 expression. J Gastroenterol Hepatol (2022) 37(1):144–53. doi: 10.1111/jgh.15657 34370878

[48] Chang IW Wu WJ Wang YH Wu TF Liang PI He HL. BCAT1 overexpression is an indicator of poor prognosis in patients with urothelial carcinomas of the upper urinary tract and urinary bladder. Histopathology (2016) 68(4):520–32. doi: 10.1111/his.12778 26173071

[49] Ananieva EA Bostic JN Torres AA Glanz HR McNitt SM Brenner MK. Mice deficient in the mitochondrial branched-chain aminotransferase (BCATm) respond with delayed tumour growth to a challenge with EL-4 lymphoma. Br J Cancer (2018) 119(8):1009–17. doi: 10.1038/s41416-018-0283-7 PMC6203766 30318512

[50] Zeng B Zhang X Zhao J Wei Z Zhu H Fu M. The role of DNMT1/hsa-miR-124-3p/BCAT1 pathway in regulating growth and invasion of esophageal squamous cell carcinoma. BMC Cancer (2019) 19(1):609. doi: 10.1186/s12885-019-5815-x 31226958 PMC6588861

[51] Guo W Wang H Li C. Signal pathways of melanoma and targeted therapy. Signal Transduct Target Ther (2021) 6(1):424. doi: 10.1038/s41392-021-00827-6 34924562 PMC8685279

[52] Luo L Sun W Zhu W Li S Zhang W Xu X. BCAT1 decreases the sensitivity of cancer cells to cisplatin by regulating mTOR-mediated autophagy *via* branched-chain amino acid metabolism. Cell Death Dis (2021) 12(2):169. doi: 10.1038/s41419-021-03456-7 33568627 PMC7876012

[53] Wang H Wang F Ouyang W Jiang X Wang Y. BCAT1 overexpression regulates proliferation and cMyc/GLUT1 signaling in head and neck squamous cell carcinoma. Oncol Rep (2021) 45(5):52. doi: 10.3892/or.2021.8003 33760210 PMC7962101

[54] Liu R Liu J Wu P Yi H Zhang B Huang W. Flotillin-2 promotes cell proliferation *via* activating the c-Myc/BCAT1 axis by suppressing miR-33b-5p in nasopharyngeal carcinoma. Aging (2021) 13(6):8078–94. doi: 10.18632/aging.202726 PMC8034900 33744853

[55] Mao L Chen J Lu X Yang C Ding Y Wang M. Proteomic analysis of lung cancer cells reveals a critical role of BCAT1 in cancer cell metastasis. Theranostics (2021) 11(19):9705–20. doi: 10.7150/thno.61731 PMC8490523 34646394

[56] Lin X Tan S Fu L Dong Q. BCAT1 overexpression promotes proliferation, invasion, and wnt signaling in non-small cell lung cancers. Onco Targets Ther (2020) 13:3583–94. doi: 10.2147/OTT.S237306 PMC7196801 32425554

[57] Mamun MA Mannoor K Cao J Qadri F Song X. SOX2 in cancer stemness: tumor malignancy and therapeutic potentials. J Mol Cell Biol (2020) 12(2):85–98. doi: 10.1093/jmcb/mjy080 30517668 PMC7109607

[58] Wang Y Zhang J Ren S Sun D Huang HY Wang H. Branched-chain amino acid metabolic reprogramming orchestrates drug resistance to EGFR tyrosine kinase inhibitors. Cell Rep (2019) 28(2):512–25. doi: 10.1016/j.celrep.2019.06.026 31291585

[59] Niraula S Le LW Tannock IF. Treatment of prostate cancer with intermittent versus continuous androgen deprivation: a systematic review of randomized trials. J Clin Oncol (2013) 31(16):2029–36. doi: 10.1200/JCO.2012.46.5492 23630216

[60] Zhu W Shao Y Peng Y. MicroRNA-218 inhibits tumor growth and increases chemosensitivity to CDDP treatment by targeting BCAT1 in prostate cancer. Mol Carcinog (2017) 56(6):1570–7. doi: 10.1002/mc.22612 28052414

[61] Chaumeil MM Larson PE Woods SM Cai L Eriksson P Robinson AE. Hyperpolarized [1-13C] glutamate: a metabolic imaging biomarker of IDH1 mutational status in glioma. Cancer Res (2014) 74(16):4247–57. doi: 10.1158/0008-5472.CAN-14-0680 PMC4134724 24876103

[62] Mayers JR Vander Heiden MG. BCAT1 defines gliomas by IDH status. Nat Med (2013) 19(7):816–7. doi: 10.1038/nm.3263 23836221

[63] Cho HR Jeon H Park CK Park SH Kang KM Choi SH. BCAT1 is a new MR imaging-related biomarker for prognosis prediction in IDH1-wildtype glioblastoma patients. Sci Rep (2017) 7(1):17740. doi: 10.1038/s41598-017-17062-1 29255149 PMC5735129

[64] Panosyan EH Lin HJ Koster J Lasky JL 3rd. In search of druggable targets for GBM amino acid metabolism. BMC Cancer (2017) 17(1):162. doi: 10.1186/s12885-017-3148-1 28245795 PMC5331648

[65] Yi L Fan X Li J Yuan F Zhao J Nistér M Yang X. Enrichment of branched chain amino acid transaminase 1 correlates with multiple biological processes and contributes to poor survival of IDH1 wild-type gliomas. Aging (2021) 13(3):3645–60. doi: 10.18632/aging.202328 PMC7906175 33493139

[66] Tonjes M Barbus S Park YJ Wang W Schlotter M Lindroth AM. BCAT1 promotes cell proliferation through amino acid catabolism in gliomas carrying wild-type IDH1. Nat Med (2013) 19(7):901–8. doi: 10.1038/nm.3217 PMC4916649 23793099

[67] Silva LS Poschet G Nonnenmacher Y Becker HM Sapcariu S Gaupel AC. Branched-chain ketoacids secreted by glioblastoma cells *via* MCT1 modulate macrophage phenotype. EMBO Rep (2017) 18(12):2172–85. doi: 10.15252/embr.201744154 PMC5709768 29066459

[68] Cho HR Hong B Kim H Park CK Park SH Choi SH. Assessment of bevacizumab resistance increased by expression of BCAT1 in IDH1 wild-type glioblastoma: application of DSC perfusion MR imaging. Oncotarget (2016) 7(43):69606–15. doi: 10.18632/oncotarget.11901 PMC5342501 27626306

[69] Anderson LC Hakansson M Walse B Nilsson CL. Intact protein analysis at 21 Tesla and X-ray crystallography define structural differences in single amino acid variants of human mitochondrial branched-chain amino acid aminotransferase 2 (BCAT2). J Am Soc Mass Spectrom (2017) 28(9):1796–804. doi: 10.1007/s13361-017-1705-0 PMC5556139 28681360

[70] Ouyang H Gao X Zhang J. Impaired expression of BCAT1 relates to muscle atrophy of mouse model of sarcopenia. BMC Musculoskelet Disord (2022) 23(1):450. doi: 10.1186/s12891-022-05332-7 35562710 PMC9102634

[71] Yoshiji H Noguchi R Kitade M Kaji K Ikenaka Y Namisaki T. Branched-chain amino acids suppress insulin-resistance-based hepatocarcinogenesis in obese diabetic rats. J Gastroenterol (2009) 44(5):483–91. doi: 10.1007/s00535-009-0031-0 19319465

[72] Yoshiji H Noguchi R Namisaki T Moriya K Kitade M Aihara Y. Branched-chain amino acids suppress the cumulative recurrence of hepatocellular carcinoma under conditions of insulin-resistance. Oncol Rep (2013) 30(2):545–52. doi: 10.3892/or.2013.2497 PMC3816550 23708326

[73] Zheng YH Hu WJ Chen BC Grahn TH Zhao YR Bao HL. BCAT1, a key prognostic predictor of hepatocellular carcinoma, promotes cell proliferation and induces chemoresistance to cisplatin. Liver Int (2016) 36(12):1836–47. doi: 10.1111/liv.13178 27246112

[74] Zhang B Xu F Wang K Liu M Li J Zhao Q. BCAT1 knockdown-mediated suppression of melanoma cell proliferation and migration is associated with reduced oxidative phosphorylation. Am J Cancer Res (2021) 11(6):2670–83. https://www.ncbi.nlm.nih.gov/pmc/articles/PMC8263658/. PMC8263658 34249421

[75] Su Y Zhao B Zhou L Zhang Z Shen Y Lv H. Ferroptosis, a novel pharmacological mechanism of anti-cancer drugs. Cancer Lett (2020) 483:127–36. doi: 10.1016/j.canlet.2020.02.015 32067993

[76] Wang K Zhang Z Tsai HI Liu Y Gao J Wang M. Branched-chain amino acid aminotransferase 2 regulates ferroptotic cell death in cancer cells. Cell Death Differ (2021) 28(4):1222–36. doi: 10.1038/s41418-020-00644-4 PMC8027606 33097833

[77] Ashby EL Kierzkowska M Hull J Kehoe PG Hutson SM Conway ME. Altered expression of human mitochondrial branched chain aminotransferase in dementia with lewy bodies and vascular dementia. Neurochem Res (2017) 42(1):306–19. doi: 10.1007/s11064-016-1855-7 PMC5283609 26980008

[78] Hull J Patel V El Hindy M Lee C Odeleye E Hezwani M. Regional increase in the expression of the BCAT proteins in alzheimer's disease brain: Implications in glutamate toxicity. J Alzheimers Dis (2015) 45(3):891–905. doi: 10.3233/JAD-142970 25633671

[79] Hull J Patel VB Hutson SM Conway ME. New insights into the role of the branched-chain aminotransferase proteins in the human brain. J Neurosci Res (2015) 93(7):987–98. doi: 10.1002/jnr.23558 25639459

[80] Li H Ye D Xie W Hua F Yang Y Wu J. Defect of branched-chain amino acid metabolism promotes the development of alzheimer’s disease by targeting the mTOR signaling. Bioscience Rep (2018) 38(4):BSR20180127. doi: 10.1042/bsr20180127 PMC6028749 29802157

[81] Hudd F Shiel A Harris M Bowdler P McCann B Tsivos D. Novel blood biomarkers that correlate with cognitive performance and hippocampal volumetry: Potential for early diagnosis of alzheimer's disease. J Alzheimers Dis (2019) 67(3):931–47. doi: 10.3233/JAD-180879 30689581

[82] Shafei MA Harris M Conway ME. Divergent metabolic regulation of autophagy and mTORC1-early events in alzheimer's disease? Front Aging Neurosci (2017) 9:173. doi: 10.3389/fnagi.2017.00173 28626421 PMC5454035

[83] Harris M El Hindy M Usmari-Moraes M Hudd F Shafei M Dong M. BCAT-induced autophagy regulates abeta load through an interdependence of redox state and PKC phosphorylation-implications in alzheimer's disease. Free Radic Biol Med (2020) 152:755–66. doi: 10.1016/j.freeradbiomed.2020.01.019 31982508

[84] Mor DE Sohrabi S Kaletsky R Keyes W Tartici A Kalia V. Metformin rescues parkinson's disease phenotypes caused by hyperactive mitochondria. Proc Natl Acad Sci U.S.A. (2020) 117(42):26438–47. doi: 10.1073/pnas.2009838117 PMC7585014 33024014

[85] Yao V Kaletsky R Keyes W Mor DE Wong AK Sohrabi S. An integrative tissue-network approach to identify and test human disease genes. HHS Public Access (2020). doi: 10.1038/nbt.4246 PMC7021177 30346941

[86] Lai Q Yuan G Shen L Zhang L Fu F Liu Z. Oxoeicosanoid receptor inhibition alleviates acute myocardial infarction through activation of BCAT1. Basic Res Cardiol (2021) 116(1):3. doi: 10.1007/s00395-021-00844-0 33484341

[87] Da Costa L Leblanc T Mohandas N. Diamond-blackfan anemia. blood (2021) 136(11):1262–73. doi: 10.1182/blood.2019000947 PMC7483438 32702755

[88] Pereboom TC Bondt A Pallaki P Klasson TD Goos YJ Essers PB. Translation of branched-chain aminotransferase-1 transcripts is impaired in cells haploinsufficient for ribosomal protein genes. Exp Hematol (2014) 42(5):394–403. doi: 10.1016/j.exphem.2013.12.010 24463277

[89] Guizar-Heredia R Tovar AR Granados-Portillo O Pichardo-Ontiveros E Flores-Lopez A Gonzalez-Salazar LE. Serum amino acid concentrations are modified by age, insulin resistance, and BCAT2 rs11548193 and BCKDH rs45500792 polymorphisms in subjects with obesity. Clin Nutr (2021) 40(6):4209–15. doi: 10.1016/j.clnu.2021.01.037 33583659

[90] Zhao X Han Q Liu Y Sun C Gang X Wang G. The relationship between branched-chain amino acid related metabolomic signature and insulin resistance: A systematic review. J Diabetes Res (2016) 2016:2794591. doi: 10.1155/2016/2794591 27642608 PMC5014958

[91] Moghei M Tavajohi-Fini P Beatty B Adegoke OA. Ketoisocaproic acid, a metabolite of leucine, suppresses insulin-stimulated glucose transport in skeletal muscle cells in a BCAT2-dependent manner. Am J Physiol Cell Physiol (2016) 311(3):C518–27. doi: 10.1152/ajpcell.00062.2016 PMC5129764 27488662

[92] Mann G Adegoke OAJ. Effects of ketoisocaproic acid and inflammation on glucose transport in muscle cells. Physiol Rep (2021) 9(1):e14673. doi: 10.14814/phy2.14673 33400857 PMC7785050

[93] Papathanassiu AE Ko JH Imprialou M Bagnati M Srivastava PK Vu HA. BCAT1 controls metabolic reprogramming in activated human macrophages and is associated with inflammatory diseases. Nat Commun (2017) 8:16040. doi: 10.1038/ncomms16040 28699638 PMC5510229

[94] Ko JH Olona A Adonia E Papathanassiu AE Buang N Park KS. BCAT1 affects mitochondrial metabolism independently of leucine transamination in activated human macrophages. JournalofCellScience (2020) 133(22):jcs247957. doi: 10.1242/jcs.247957 PMC7116427 33148611

[95] Lang CH Lynch CJ Vary TC. BCATm deficiency ameliorates endotoxin-induced decrease in muscle protein synthesis and improves survival in septic mice. Am J Physiol Regul Integr Comp Physiol (2010) 299(3):R935–44. doi: 10.1152/ajpregu.00297.2010 PMC2944428 20554928

[96] Pereira S Ley D Schubert M Grav LM Kildegaard HF Andersen MR. BCAT1 and BCAT2 disruption in CHO cells has cell line-dependent effects. J Biotechnol (2019) 306:24–31. doi: 10.1016/j.jbiotec.2019.08.017 31465797

[97] Mulukutla BC Mitchell J Geoffroy P Harrington C Krishnan M Kalomeris T. Metabolic engineering of Chinese hamster ovary cells towards reduced biosynthesis and accumulation of novel growth inhibitors in fed-batch cultures. Metab Eng (2019) 54:54–68. doi: 10.1016/j.ymben.2019.03.001 30851381

[98] Chen S Chen B Su G Chen J Guo D Yin Q. Branched-chain amino acid aminotransferase-1 regulates self-renewal and pluripotency of mouse embryonic stem cells through ras signaling. Stem Cell Res (2020) 49:102097. doi: 10.1016/j.scr.2020.102097 33271468

[99] Grankvist N Lagerborg KA Jain M Nilsson R. Gabapentin can suppress cell proliferation independent of the cytosolic branched-chain amino acid transferase 1 (BCAT1). Biochemistry (2018) 57(49):6762–6. doi: 10.1021/acs.biochem.8b01031 PMC6528808 30427175

[100] Bertrand SM Ancellin N Beaufils B Bingham RP Borthwick JA Boullay AB. The discovery of *in vivo* active mitochondrial branched-chain aminotransferase (BCATm) inhibitors by hybridizing fragment and HTS hits. J Med Chem (2015) 58(18):7140–63. doi: 10.1021/acs.jmedchem.5b00313 26090771

[101] Borthwick JA Ancellin N Bertrand SM Bingham RP Carter PS Chung CW. Structurally diverse mitochondrial branched chain aminotransferase (BCATm) leads with varying binding modes identified by fragment screening. J Med Chem (2016) 59(6):2452–67. doi: 10.1021/acs.jmedchem.5b01607 26938474

[102] Li GS Huang HQ Liang Y Pang QY Sun HJ Huang ZG. BCAT1: A risk factor in multiple cancers based on a pan-cancer analysis. Cancer Med (2022) 11(5):1396–412. doi: 10.1002/cam4.4525 PMC8894718 34984849

