# Supplementary material for: The mechanism of branched-chain amino acid transferases in different diseases: Research progress and future prospects
Source: Front Oncol. 2022 Sep 2;12:988290. doi: 10.3389/fonc.2022.988290 (PMC9478667; doi:10.3389/fonc.2022.988290)
Supplement: Supplementary file 1 [file DataSheet_1.docx]

**Supplementary Materials**

The protein sequences of human, mouse, and rat BCAT1-2 from the Unified Protein Data Bank are described as follows.

**1. Branched-chain-amino-acid aminotransferase, cytosolic · Homo sapiens (Human)**

**2. Branched-chain-amino-acid aminotransferase, cytosolic · Rattus norvegicus (Rat)**

**3. Branched-chain-amino-acid aminotransferase, cytosolic · Mus musculus (Mouse)**

**4. Branched-chain-amino-acid aminotransferase, mitochondrial · Homo sapiens (Human)**

**5. Branched-chain-amino-acid aminotransferase, mitochondrial · Rattus norvegicus (Rat)**

**6. Branched-chain-amino-acid aminotransferase, mitochondrial · Mus musculus (Mouse)**

**1. Branched-chain-amino-acid aminotransferase, cytosolic · Homo sapiens (Human)**

**amino acid sequence:**

>sp|P54687|BCAT1_HUMAN Branched-chain-amino-acid aminotransferase, cytosolic OS=Homo sapiens OX=9606 GN=BCAT1 PE=1 SV=3

MKDCSNGCSAECTGEGGSKEVVGTFKAKDLIVTPATILKEKPDPNNLVFGTVFTDHMLTV

EWSSEFGWEKPHIKPLQNLSLHPGSSALHYAVELFEGLKAFRGVDNKIRLFQPNLNMDRM

YRSAVRATLPVFDKEELLECIQQLVKLDQEWVPYSTSASLYIRPTFIGTEPSLGVKKPTK

ALLFVLLSPVGPYFSSGTFNPVSLWANPKYVRAWKGGTGDCKMGGNYGSSLFAQCEAVDN

GCQQVLWLYGEDHQITEVGTMNLFLYWINEDGEEELATPPLDGIILPGVTRRCILDLAHQ

WGEFKVSERYLTMDDLTTALEGNRVREMFGSGTACVVCPVSDILYKGETIHIPTMENGPK

LASRILSKLTDIQYGREESDWTIVLS

**Human BCAT 1 :**

**Seq Identity**

99.74%

Template

2cog.1.A branched chain aminotransferase 1, cytosolic

Crystal structure of oxidized human cytosolic branched-chain aminotransferase complexed with 4-methylvalerate

**2. Branched-chain-amino-acid aminotransferase, cytosolic · Rattus norvegicus (Rat)**

**amino acid sequence :**

>sp|P54690|BCAT1_RAT Branched-chain-amino-acid aminotransferase, cytosolic OS=Rattus norvegicus OX=10116 GN=Bcat1 PE=1 SV=1

MAYLSRATATLARQDCSNGCSASYAEEEELEASTESYDEEGGSEASTQTFRAKDLIITKA

DVLKKKPDPSSLVFGASFTDHMLMVEWTSKYGWDKPHIKPFENLSIHPAASVLHYAVELF

EGLKAFRGVDNKIRLFRPDLNMKRMCRSAVRTTLPEFDKEELLQCVLQLIQLDREWVPYS

TSASLYIRPTFIGIEPSLGVKKPSKALLFVILSPVGSYFSNGTFSPVSLWANPKFVRSWK

GGTGDFKMGCNYGSSLLAQCEAAENGCHQVLWLYGKENRITEVGTMNLFLYWINKDGEEE

LATPPLDGVILPGVTRQSILELGEEWGEFKVCERHITMDDLSTALEENRVKEMFGSGTAC

VVCPVASILYKGQMLHIPTMENGHKLSSRIMAKLTDIQYGRIKSEWTLELP

**Rat BCAT 1 :**

Seq Identity

76.17%

Template

2cog.1.A branched chain aminotransferase 1, cytosolic

Crystal structure of oxidized human cytosolic branched-chain aminotransferase complexed with 4-methylvalerate

**3. Branched-chain-amino-acid aminotransferase, cytosolic · Mus musculus (Mouse)**

**amino acid sequence :**

>sp|P24288|BCAT1_MOUSE Branched-chain-amino-acid aminotransferase, cytosolic OS=Mus musculus OX=10090 GN=Bcat1 PE=1 SV=2

MKDCSNGCSAPFAGERGSEEVAETFRAKDLIITPATVLKEKPDPDSLVFGATFTDHMLTV

EWSSASGWEKPHIKPFGNLPIHPAASVLHYAVELFEGLKAFRGVDNKIRLFRPDLNMDRM

CRSAVRTTLPMFDKEELLKCILQLLQIDQEWVPYSTSASLYIRPTFIGTEPSLGVKKPSK

ALLFVILSPVGPYFSSGSFTPVSLWANPKYIRAWKGGTGDCKMGGNYGASLLAQCEAVEN

GCQQVLWLYGKDNQITEVGTMNLFLYWINEDGEEELATPPLDGIILPGVTRQSILELAQQ

WGEFKVCERHLTMDDLATALEGNRVKEMFGSGTACVVCPVSDILYKGQMLHIPTMENGPK

LASRILGKLTDIQYGRVESDWTIELP

**Mouse BCAT 1 :**

Seq Identity

84.94%

Template

2cog.1.A branched chain aminotransferase 1, cytosolic

Crystal structure of oxidized human cytosolic branched-chain aminotransferase complexed with 4-methylvalerate

**4. Branched-chain-amino-acid aminotransferase, mitochondrial · Homo sapiens (Human)**

**amino acid sequence :**

>sp|O15382|BCAT2_HUMAN Branched-chain-amino-acid aminotransferase, mitochondrial OS=Homo sapiens OX=9606 GN=BCAT2 PE=1 SV=2

MAAAALGQIWARKLLSVPWLLCGPRRYASSSFKAADLQLEMTQKPHKKPGPGEPLVFGKT

FTDHMLMVEWNDKGWGQPRIQPFQNLTLHPASSSLHYSLQLFEGMKAFKGKDQQVRLFRP

WLNMDRMLRSAMRLCLPSFDKLELLECIRRLIEVDKDWVPDAAGTSLYVRPVLIGNEPSL

GVSQPTRALLFVILCPVGAYFPGGSVTPVSLLADPAFIRAWVGGVGNYKLGGNYGPTVLV

QQEALKRGCEQVLWLYGPDHQLTEVGTMNIFVYWTHEDGVLELVTPPLNGVILPGVVRQS

LLDMAQTWGEFRVVERTITMKQLLRALEEGRVREVFGSGTACQVCPVHRILYKDRNLHIP

TMENGPELILRFQKELKEIQYGIRAHEWMFPV

**Human BCAT 2 :**

Seq Identity

100.00%

Template

5cr5.1.A Branched-chain-amino-acid aminotransferase, mitochondrial

X-RAY CRYSTAL STRUCTURE AT 1.61A RESOLUTION OF HUMAN MITOCHONDRIAL BRANCHED CHAIN AMINOTRANSFERASE (BCATM) COMPLEXED WITH A BIPHENYL PYRROLIDINE ETHER COMPOUND AND AN INTERNAL ALDIMINE LINKED PLP COFACTOR.

**5. Branched-chain-amino-acid aminotransferase, mitochondrial · Rattus norvegicus (Rat)**

**amino acid sequence :**

>sp|O35854|BCAT2_RAT Branched-chain-amino-acid aminotransferase, mitochondrial OS=Rattus norvegicus OX=10116 GN=Bcat2 PE=1 SV=1

MSAAILGQVWTRKLLPIPWRLCVPGRCVSSNFKAADLQVQVTREPQKKPAPSQPLLFGKT

FTDHMLMVEWNSKTGWGPPRIQPFQNLTLHPACSGLHYSLQLFEGLKAYKGRDKQVRLFR

PWLNMDRMLRSARRLCLPDFDKQELLECIRQLIEVDKDWVPDGNGTSLYVRPVLIGNEPS

LGVGMVTQALLFVILCPVGSYFPGDSMTPVSLLADPSFVRAWIGGVGDCKLGGNYGPTVA

VQQEAQKKGCEQVLWLYGPDHQLTEVGTMNIFVYWTHEDGELELATPPLDGIILPGVVRQ

SLLDLARTWGEFRVAERKVTMKELKRALEEGRVREVFGSGTACQVCPVHQILYEGKQLHI

PTMENGPELILRFQKELKAIQYGTSAHDWMLRV

**Rat BCAT 2 :**

Seq Identity

82.47%

Template

5i5u.1.A Branched-chain-amino-acid aminotransferase, mitochondrial

X-RAY CRYSTAL STRUCTURE AT 2.40A RESOLUTION OF HUMAN MITOCHONDRIAL BRANCHED CHAIN AMINOTRANSFERASE (BCATM) COMPLEXED WITH A TETRAHYDRONAPHTHALENYL COMPOUND AND AN INTERNAL ALDIMINE LINKED PLP

**6. Branched-chain-amino-acid aminotransferase, mitochondrial · Mus musculus (Mouse)**

**amino acid sequence :**

>sp|O35855|BCAT2_MOUSE Branched-chain-amino-acid aminotransferase, mitochondrial OS=Mus musculus OX=10090 GN=Bcat2 PE=1 SV=2

MAAATLGQVWARKLLPVPWLLCGSKRCVSSIFKAADLQIQMTKEPQKKPAPSQALLFGKT

FTDHMLMVEWNNKAGWGPPRIQPFQNLTLHPACSGLHYSLQLFEGLKAYKGGDQQVRLFR

PWLNMDRMLRSARRLCLPDFDKQELLECIRQLIEVDKDWVPDGNGTSLYVRPVLIGNEPS

LGVGMVTQALLYVILCPVGSYFPGDSMTPVSLLADPSFVRAWIGGVGDCKLGGNYGPTVA

VQREAQKRGCEQVLWLYGPDHQLTEVGTMNIFVYWTHEDGVLELVTPPLNGVILPGVVRQ

SLLDLARTWGEFRVAERKVTMKELKRALEEGRVREVFGSGTACQVCPVHQILYEGKQLHI

PTMENGPELILRFQKELKAIQYGASAHDWMFRV

**Mouse BCAT 2 :**

Seq Identity

83.84%

Template

5cr5.1.A Branched-chain-amino-acid aminotransferase, mitochondrial

X-RAY CRYSTAL STRUCTURE AT 1.61A RESOLUTION OF HUMAN MITOCHONDRIAL BRANCHED CHAIN AMINOTRANSFERASE (BCATM) COMPLEXED WITH A BIPHENYL PYRROLIDINE ETHER COMPOUND AND AN INTERNAL ALDIMINE LINKED PLP COFACTOR.
